# A magnetically actuated microcatheter with soft rotatable tip for enhanced endovascular access and treatment efficiency

**DOI:** 10.1126/sciadv.adv1682

**Published:** 2025-06-20

**Authors:** Moqiu Zhang, Lidong Yang, Haojin Yang, Lin Su, Junnan Xue, Qinglong Wang, Bo Hao, Yihang Jiang, Kai Fung Chan, Joseph Jao Yiu Sung, Ho Ko, Xurui Liu, Liu Wang, Bonaventure Yiu Ming Ip, Thomas Wai Hong Leung, Li Zhang

**Affiliations:** ^1^ Department of Mechanical and Automation Engineering, The Chinese University of Hong Kong, Hong Kong, SAR 999077, P.R. China.; ^2^ Research Institute for Advanced Manufacturing, Department of Industrial and Systems Engineering, The Hong Kong Polytechnic University, Hong Kong, China.; ^3^ Chow Yuk Ho Technology Centre for Innovative Medicine, The Chinese University of Hong Kong, Shatin, N.T., Hong Kong, China.; ^4^ Li Ka Shing Institute of Health Sciences, The Chinese University of Hong Kong, Hong Kong SAR, China.; ^5^ Lee Kong Chian School of Medicine, Nanyang Technological University, Singapore, Singapore.; ^6^ Department of Medicine and Therapeutics, The Chinese University of Hong Kong, Shatin, N.T., Hong Kong, China.; ^7^ Department of Modern Mechanics, University of Science and Technology of China, Hefei, China.; ^8^ Gerald Choa Neuroscience Institute, Faculty of Medicine, the Chinese University of Hong Kong, Shatin, N.T., Hong Kong, China.; ^9^ Li Ka Shing Health Science Institute, Faculty of Medicine, the Chinese University of Hong Kong, Shatin, N.T., Hong Kong, China.; ^10^ Department of Surgery, The Chinese University of Hong Kong, Shatin, N.T., Hong Kong, China.

## Abstract

Endovascular interventions require fast access to affected regions, followed by effective treatment. Catheterizations are effective approaches for treating vascular diseases; however, they face challenges in accessibility, efficiency, and invasiveness in narrow, tortuous vascular systems. This study presents a submillimeter magnetically actuated soft rotatable-tipped microcatheter (MSRM) designed to access small blood vessels and provide efficient, minimally invasive therapeutic interventions for blood clot treatment. The MSRM’s rotatable tip design enhances accessibility and navigation speed through a rotation-assisted active steering strategy. Improved blood clot treatment efficiency is achieved through the MSRM’s multifunctionality: It can accelerate drug-blood clot interactions, mechanically break down blood clots, and retrieve clot debris. The low invasiveness is attributed to the soft material design and conservative actuation strategy. The performance of the MSRM is validated in both in vitro phantom studies and in vivo rabbit models, and the invasiveness is evaluated using a human placenta model.

## Teaser

A soft magnetically actuated rotor-tipped microcatheter enhances navigation and efficiently treats various types of blood clots.

## INTRODUCTION

Vascular diseases affecting deep-seated organs in the human body are serious medical conditions that require prompt access to appropriate medical devices and effective interventions. Failure to address these issues in a timely manner can lead to severe consequences. For instance, untreated endovascular blockage in cerebral vessels can lead to an irreversible loss of nervous tissue, equivalent to the brain aging 3.6 years/hour (*1*). Delayed treatment of myocardial infarction can increase the risk of 1-year mortality by 7.5% for every 30 min (*2*).

Mechanical thrombectomy (MT), which physically removes or retrieves blood clots, provides prompt and effective endovascular treatment for large vessel diseases. Recent studies have shown that MT is becoming increasingly feasible for distal medium vessel occlusions (*3*, *4*). However, conventional passively navigated interventional tools still face several limitations, especially in anatomically complex areas such as the M3 and M4 segments of the middle cerebral artery (MCA) and the distal segments of the coronary arteries (*5*). First, the absence of active steerability compromises procedural precision, leading to prolonged operation times, suboptimal device placement, and poorer patient outcomes (*6*–*11*). Second, conventional interventional tools are single-function devices, requiring repeated exchanges between navigational and therapeutic tools during operation (*12*). Frequent device exchanges pose risks to smaller vessels, elevating susceptibility to perioperative complications (*13*).

In recent years, magnetic microrobots designed for medical tasks have provided feasible solutions in otherwise challenging clinical scenarios, such as the delivery of therapeutic drugs to cancer cell target lesions, the noninvasive diagnosis through miniaturized sensors and actuators, and the endovascular recanalization of occluded arteries (*14*–*23*). Robotic magnetically actuated microcatheters, another type of magnetic microrobots, integrate magnetic actuation with microcatheter technology to facilitate effective and efficient endovascular interventions (*24*–*26*). The magnetic force and torque can be directly applied to the distal end, allowing it to maintain high maneuverability at small scales (*27*, *28*). The integration of proximal rotation and magnetic field guidance would further enhance steering performance and accessibility. A screw-tip magnetically steerable needle is proposed to drill through biological tissue via torque transmitted from the proximal end (*29*). Using the same method for generating rotation, researchers developed a magnetically steerable microcatheter with a helical surface (*30*). This design is intended to engage with the blood vessel wall, enhancing accessibility by converting rotational torque into axial motion. In addition, magnetically actuated microcatheters, compared to tendon- or hydraulically driven ones, provide more on-board space to enhance functional developments (*31*). Beyond connecting the interior and exterior of the human body and active steerability, researchers have equipped the magnetic microcatheter with sensing modules that enable the measurement of endovascular biological information, such as vessel occlusion and temperature (*27*, *32*). Furthermore, the integration of actuation modules has been demonstrated for rotational atherectomy and capsule delivery (*16*, *33*, *34*).

Despite substantial progress in recent years, applying microcatheters in small-scale blood vessels still presents notable challenges. The first issue is how to maintain high flexibility in the complex vascular system. Precurved guidewires can lose their steerability due to continuous sharp turns and singular configurations such as looping and buckling (*35*). Upon reaching the affected region, how to deliver efficient and effective therapy poses the second challenge. Limiting the tool size to the vascular scale leads to higher fabrication complexities and lower medication-target interaction efficiency (*33*, *36*). Third, the potential endothelial damage from the physical interaction between rigid actuation units and fragile blood vessels should be cautiously tabulated, particularly in terminal arteries that are prone to avulsion or rupture (*37*). The delicate nature of the soft biological material encumbers the utilization of aggressive mechanical interactions, which was once useful in large vessel treatments (*12*, *13**,* *23*).

**Fig. 1.**
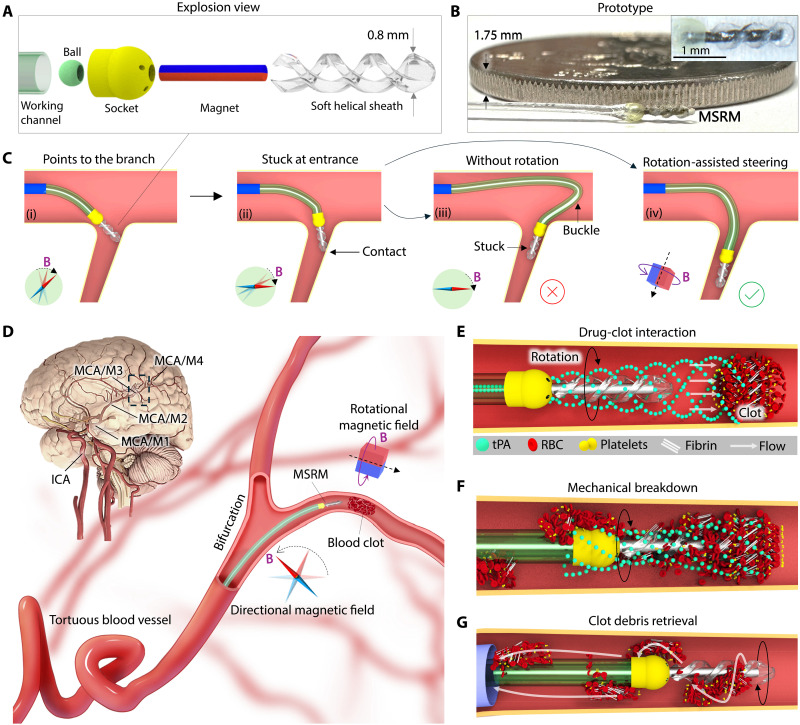
Design of the magnetic microcatheter system for endovascular treatments. (**A**) Exploded view of the microcatheter design. (**B**) MSRM prototype that is compared to a one-dollar Hong Kong dollar coin. The inset shows the microscopic view. (**C**) Schematic illustration of the rotation-assisted steering strategy and its advantages. (i to iii) Convention method to navigate the microcatheter to the sharp turn branch where the friction on the tip could cause the catheter to buckle. (iv) Rotational motion at the tip releases the contact friction at the tip and smooths the insertion process. (**D**). Schematic diagram of the magnetically actuated microcatheter system working principle. The magnetically actuated microcatheter can efficiently navigate through narrow blood vessels and generate therapeutic interventions from three aspects: drug-clot interaction acceleration (**E**), rotational mechanical breakdown (**F**), and clot debris retrieval (**G**).

To address the challenges mentioned above, we present a submillimeter-scale, soft-tipped magnetic microcatheter for navigating complex vascular systems and delivering efficient endovascular interventions. The miniaturized ball joint enables rotation along both the axial and radial axes, allowing for three-dimensional (3D) rotational motion through sequential magnetic actuation while maintaining a fluid pathway between internal and external environments (Fig. 1, A and B, and fig. S1). The axial rotation enhances the accessibility to hard-to-reach branches by reducing contact friction at the tip, avoiding potential buckling and looping scenarios. Taking advantage of this, a rotation-assisted active steering strategy is proposed (Fig. 1C), and a maximum 50% reachable workspace enhancement is experimentally verified in the Results section (Rotation-assisted active steering). Furthermore, the magnetically actuated soft rotatable-tipped microcatheter (MSRM) enables multiple therapeutic functions, including enhanced drug-thrombus interaction, blood clot mechanical breakdown, and clot debris retrieval (Fig. 1, D to G). This all-in-one design provides an effective method to reduce operational duration and improve the overall efficiency of blood clot treatments. The rotatable helical tip, made of low-stiffness elastomer, mitigates the risk of vascular damage during navigation and mechanical blood clot removal. Unlike previously reported rigid spinners that use aggressive rotating motion (*12*, *23*), the proposed microcatheter could realize efficient treatment without using large contact forces and high rotational speed. The MSRM’s high effectiveness and low invasiveness are experimentally verified in human cerebral vascular silicone phantoms, in an in vivo rabbit model, and in an ex vivo human placenta model.

## RESULTS

### Magnetic microcatheter system design

The proposed magnetic microcatheter is designed for navigating vascular systems with a diameter of 1 mm or larger, such as cortical branch arteries in the M2 and M3 segments or transition bifurcations from the M3 to M4 segments of the MCA (*38*). To meet the requirements of accessibility and invasiveness of these regions, we proposed a submillimeter robotic microcatheter design, as shown in Fig. 1A. The magnetically steerable tip includes a helical-shaped elastomer sheath, a cylindrical permanent magnet, and a miniaturized ball joint that provides two controllable rotational degrees of freedom. The bearing chamber is installed on a flexible hollow working channel, which provides pushability and fluid delivery. With its largest outer diameter (OD) designed to be 800 μm, the device is comparable in scale to a neurovascular guidewire (Nitrex Nitinol Guidewire, Medtronic). The prototype is illustrated in Fig. 1B, and the detailed dimensions are presented in table S1. The proposed microcatheter can be regarded as a steerable magnetic guidewire that can navigate sharp corners in the blood vessels (Fig. 1C) and can also be regarded as a micropump for drug-clot interaction acceleration, a spinning tool to conduct mechanical breakdown and a retrieval tool for capturing blood clot debris to the support catheter (Fig. 1G). At the proximal end, a syringe pump and a vacuum pump are connected to the working channel and the support sleeve, respectively, to enable fluid delivery and suction.

### Rotation-assisted active steering

**Fig. 2.**
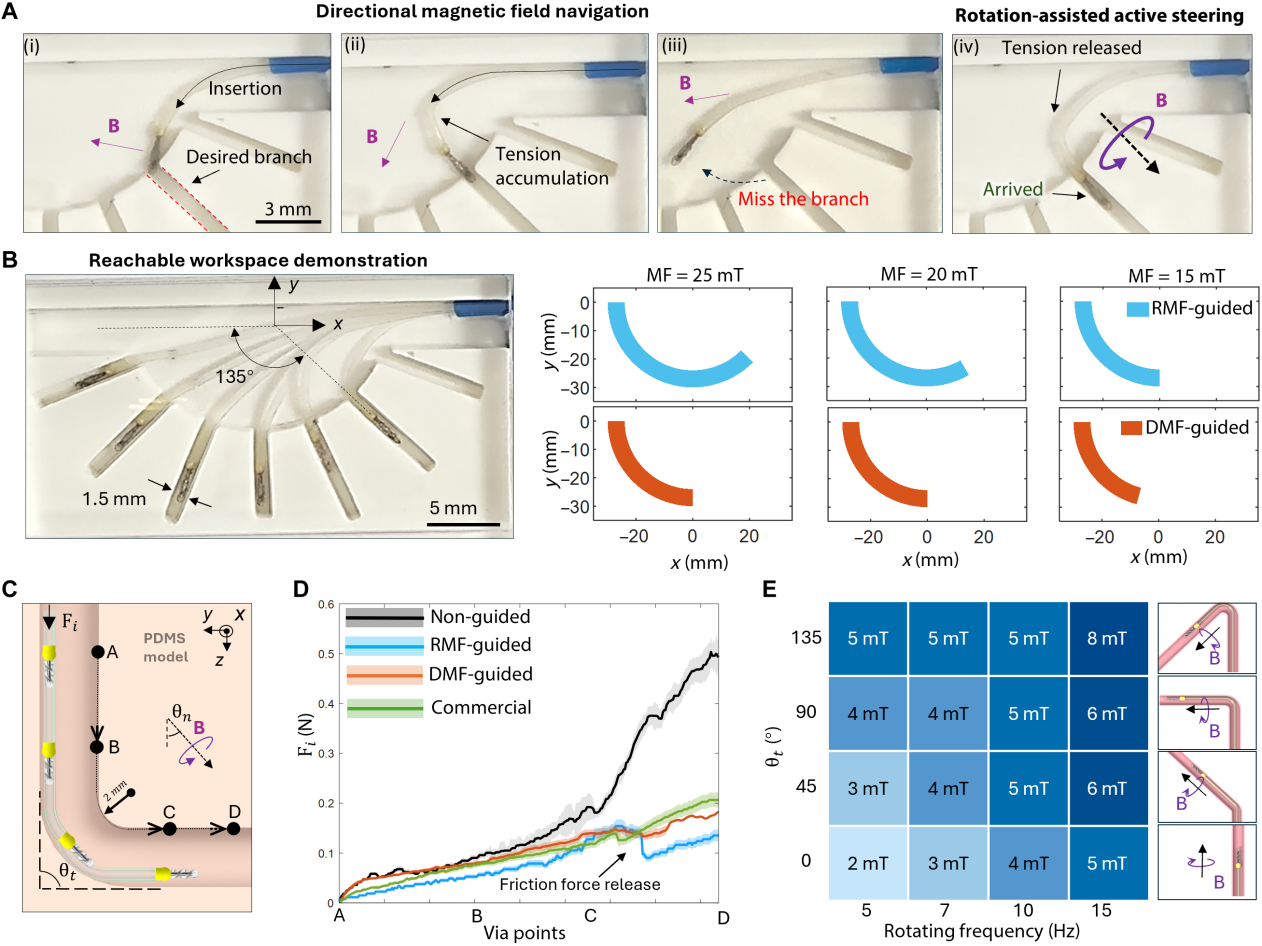
Rotation-assisted active steering strategy. (**A**) Key experiment frames of using rotation-assisted steering strategy to enter the sharp-turn branch. (i and ii) The directional field guides the microcatheter’s tip to the entrance. The stuck at the tip results in tension accumulation on the body of the catheter. (iii) Further advancement caused failure to enter the branch. (iv) Rotation motion decreases the friction force at the tip and directs the microcatheter to the desired branch. The magnetic field directions are marked by the purple arrows. (**B**) Keyframes of the experiment using the DMF to guide the microcatheter to the bifurcation phantom and the reachable workspace comparison between the DMF steering method (DMF-guided) and RMF-guided steering method (RMF-guided). A maximum 50% reachable workspace enhancement is observed at a magnetic field of 25 mT. Color areas represent the bending range of the microcatheter in the branch phantom. (**C**) Insertion force comparisons between passive insertion and rotation-assisted insertion through navigating a sharp turn. The L-shaped channel is divided into three segments by the point from A to D. In the rotation-assisted group, the norm direction of the rotation field $\theta_{n}$ is determined by the trajectory tangent direction. The branch angle $\theta_{t}$ = 90° describes the sharpness of the corner. (**D**) Results of the insertion force comparison that shows the RMF-guided group requires the least force compared to the DMF-guided group, commercial guidewire group, and nonguided group. The force reduction in segment C to D highlights friction release due to rotational motion, with color bands representing SD and lines depicting mean values. Each group of tests is repeated five times. (**E**) Required RMF strength when the microcatheter is in various branch angles.

When navigating microcatheters from the trunk vascular system to narrow collateral branches, the precurved or magnetically guided tip often encounters frictional resistance at the entrance. This resistance may lead to buckling or misdirection upon further advancement of the microcatheter, as shown in Fig. 1C. We address this issue by releasing the accumulated contact force through rotational actuation. The proposed steering strategy includes three steps: First, we use a directional magnetic field (DMF) to align the microcatheter tip with the desired branch. Subsequently, the microcatheter advances, allowing the tip to reach the branch entrance. Last, a rotating magnetic field is applied to guide the microcatheter to the desired branch and avoid buckling or looping due to rotational contact with the blood vessel wall. Figure 2A shows the failure case using the directional field to guide the microcatheter and the successful case using the proposed steering strategy. Figure 2B shows the keyframes of the experimental demonstration using the proposed navigation strategy in a bifurcation phantom. The system setup is detailed in fig. S2A. The vascular phantom was placed above a robotic arm–assisted magnetic actuation system, consisting of a 5–degrees-of-freedom robotic arm and a motorized permanent magnet. A camera tracked the position of the microrobot in real time, allowing the host computer to generate feedback-controlled magnetic fields. We further characterize the effect of magnetic field strength on steerability enhancement. The experimental results show that the rotation-assisted method was able to enlarge the reachable workspace under varying magnetic fields and realize a maximum 50% increment under a magnetic field strength of 25 mT. A detailed navigation demonstration is provided in movie S1.

In addition to improving steerability, rotational motion can enhance navigation efficiency when passing through sharp turns. Conventional catheters typically rely on the contact force between the tip and the blood vessel wall to passively change the catheter’s body shape and tip orientation, conforming to the shape of the blood vessel. The friction and contact forces increase throughout this process, posing a potential risk of insertion failure or tissue puncture. The rotation-assisted steering method can mitigate this adverse effect by the rotatable design. We validate this advantage by experimentally comparing the insertion forces required for navigating through an L-shaped channel under four different cases: with DMF guidance (DMF-guided), with rotational magnetic field navigation (RMF-guided), without magnetic field guidance (nonguided), and with commercially available guidewire (Boston Scientific, TRANSEND EX Guidewire, 0.41 mm in diameter). Figure 2C shows the schematic experiment settings, and fig. S3 shows the experiment setup. The microcatheter was fixed to the tensile measurement system (MACH-1) and inserted along the positive *z* axis direction into the curved polydimethylsiloxane (PDMS) model at a speed of 0.1 mm/s. The model was filled with glycerol fluid to simulate the viscosity and friction coefficient of blood. The insertion force was recorded to characterize the degree of navigation difficulty between different navigation strategies, and the results are presented in Fig. 2D.

Compared with the other cases, the RMF-guided strategy decreased the insertion force in different sections with adaptable mechanisms. During navigation in the straight channel, e.g., from point A to B, keeping the catheter tip centered within the lumen to avoid contact with the wall is difficult. This contact can lead to excess friction, increasing the necessary insertion force and potentially causing damage to the vessel wall. The RMF-guided microcatheter contacted the blood vessel wall with its soft rotating tip, which converts slide friction to rotation friction, reducing the navigation resistance. From point A to B, $F_{i}$ of the RMF-guided case is the lowest and the most stable one. Navigation from point B to C is to simulate the condition of the catheter passing through a sharp turn. The nonguided case shows a substantial increase in insertion force due to the accumulated friction. For the RMF-guided case, the $F_{i}$ first fluctuated due to the rotation constantly releasing the accumulated friction. When the contact and friction are large enough to stop the rotation, the magnetic torque continues to provide both bending and propulsive force. Navigation from point C to D is to simulate the condition of the catheter after it passes through the curved lumen. With the rotation resumed, the decrease in the insertion force is observed due to friction force release at the tip, as highlighted by the black arrow in Fig. 2D. It is worth noting that the tip rotation also detached the catheter body from the blood vessel wall, thus alleviating the friction of the entire distal end. Experiment results suggest that the proposed method reduces maximum insertion force by 69, 18, and 25% compared to the nonguided group, DMF-guided group, and commercial group, respectively. A detailed navigation test is presented in movie S2.

As the microcatheter is advanced into bifurcated branch vessels, it faces both external friction from the vessel wall and internal friction from the ball joint. The successful magnetic rotation of the tip under these resistive conditions, using a practical magnetic field strength, will be pivotal for its effectiveness in clinical applications. To validate the catheter’s ability to rotate under these resistive conditions, we conducted experiments using a 3D-printed vascular phantom rinsed (fig. S4) with glycerol to simulate blood vessel wall friction [~0.2, consistent with clinical interactions (*39*)]. The microcatheter was inserted into the branch with different bending angles, and RMFs (1 to 10 mT, 1 to 8 Hz) were applied—parameters spanning our system’s operational range and clinically feasible settings. Although phase differences between the field and tip orientation were not explicitly measured, rotation consistency was monitored through frame-by-frame video analysis. The minimum actuation field is recorded and presented in Fig. 2E. The rotatable tip can be successfully actuated by an RMF with strengths ranging from 2 to 8 mT, even in large bending angles (135°). This magnetic field requirement can be met by several clinically feasible magnetic actuation systems (*14*, *30*), which suggests its clinical application potential.

### Structural optimization for enhanced blood clot treatment

**Fig. 3.**
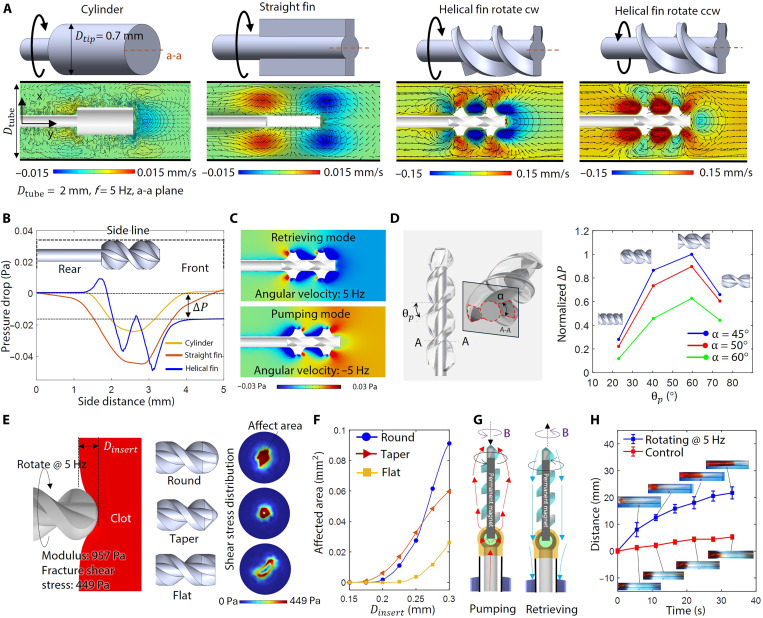
Rotational actuation design and simulation results. (**A**) Different tip designs: cylinder, straight fins, and helical fins with different rotation directions. (**B**) Side edge pressure distribution with respect to the distance to the catheter’s rear end at 5 Hz. (**C**) Pressure distribution of retrieving mode and pumping mode. (**D**) Simulation results to optimize the structural design of the rotatable tip. The optimal values are $\theta_{p}$ = 60° and α = 45°. (**E**) Simulation settings to investigate the optimal tip profile. (**F**) Simulation result that shows the affected area of different tip profile designs. (**G**) Pumping effect comparing with natural diffusion at rotation velocity of 5 Hz. Each group of tests is repeated three times. (**H**) Schematic flow status of the pumping mode and retrieving mode.

The MSRM’s pumping effect and blood clot debris-retrieving ability are attributed to the directional flow induced by rotational motion and the pressure drop between the rear and front ends. Computational fluid dynamics (CFD) is used to investigate the hydrodynamics of various rotatable tip designs to achieve optimal performance. Starting with a rotating microcylinder, the most intuitive design for generating flow, we analyze the flow velocity field and pressure distribution. As shown in Fig. 3A, a rotating cylinder (5 Hz) inside a tube generates localized negative pressure that attracts surrounding fluids, as indicated by the flow velocity direction (black arrows) on the a-a plane toward the microrotor. In the second design, we incorporate straight fins to increase the pressure drop by facilitating greater flow. The two evenly spaced straight fins can achieve nearly 10 times the flow velocity. The fluid flow rate can be further enhanced and made reversible by adding helical fins, as illustrated in the third design in Fig. 3A. This is evidenced by both high flow velocity and a larger pressure drop between the rear and front of the microrotor, as shown in Fig. 3 (B and C). Unlike the cylinder and straight fins designs, which tend to retain clot debris on the rotor, the helical fins design creates a pressure difference between the rear and front of the microrotor. The resulting backward flow carries the debris to the supporting aspiration catheter.

Another function of the helical design is to generate thrust flow and pressurize the surrounding fluid. As shown in the fourth design in Fig. 3A, by switching the rotation direction, the microrotor can transition from retrieval mode to pumping mode. This pumping effect is critical for mixing the thrombolytic drug with the blood clot. The fluid environment in small-scale vessels is characterized by low Reynolds numbers (≤0.1), which leads to slow fluid diffusion and inefficient suction force (*40*). Actively generating directional flow to pump the fluid to the desired region and increasing the blood clot–drug mixing rate can overcome the challenges of diffusion. The pumping effect is optimized by achieving the best combination of critical parameters through the CFD simulation. Similar to an axial flow pump, the helical pitch angle ( $\theta_{p}$ ) and the fin’s central angle (α) are two key variables influencing the pumping efficiency, as shown in Fig. 3D. The simulation results showed that the optimal performance is achieved at $\theta_{p}$ = 60°, with fluid pumping performance increasing with the central angle α. To ensure good structural integrity for effective pumping, α = 45° is adopted for the subsequent studies. Simulation tests are conducted to verify the structural integrity of the fins during rotation, as shown in fig. S5.

The MSRM’s clot-breaking mechanism is attributed to the shear force applied to the clot. The rotating microrotor contacts the blood clot and generates a continuous shear force to break down the coagulation of red blood cells. The contact region where the shear stress exceeds the shear stress of the blood clot fraction is deemed as the affected area (*41*). Computer-aided engineering (CAE) is used to investigate the optimal tip profile for maximizing the affected area when the tip of the MSRM is rotated and inserted into the blood clot, as illustrated in Fig. 3E. Three tip profile designs—round, tapered, and flat—are tested, with each design resulting in different shear stress distributions, as shown on the right side. More details regarding the simulations can be found in the Materials and Methods section (MSRM and blood clot contact simulation). Figure 3F presents simulation results indicating that, when the tip is inserted into the blood clot, the affected area of the round tip design is nearly 130% of that of the tapered design and 300% of that of the flat design. Therefore, here, we selected the round tip design.

Guided by the simulation results, we fabricated the prototype of the microcatheter (Fig. 1B). Upon rotating magnetic field actuation, the helical tip propels fluid forward or induces a backward retrieval flow to the aspiration catheter (Fig. 3G). We experimentally verify this “pumping effect,” with details provided in fig. S6A. The microcatheter generated thrust flow to propel the red dye uphill in a silicone tubular model, overcoming the gravitational pressure barrier. The movement of the red dye boundary served as a measure of performance. The results in Fig. 3H verify the pumping mode’s performance. Upon reaching the midpoint, the MSRM could increase the red dye boundary moving speed by nearly 10 times compared to the control group. A detailed demonstration can be found in movie S3.

### In vitro navigation demonstration

Manually rotating the proximal end allows for the steering of a precurved guidewire or microcatheter during branch selection. However, after navigating through multiple turns, the torque at the proximal end becomes limited, which restricts control efficiency. In addition, maneuvering the catheter tip through sharp turns—such as entering a recurrent branch vessel that originates at an angle greater than 90° to the parent vessel or navigating multiple consecutive sharp turns with a limited curvature radius—presents notable challenges. We conducted experiments in a full-size human brain vessel phantom to validate the proposed microcatheter’s advantage in addressing these navigation difficulties.

**Fig. 4.**
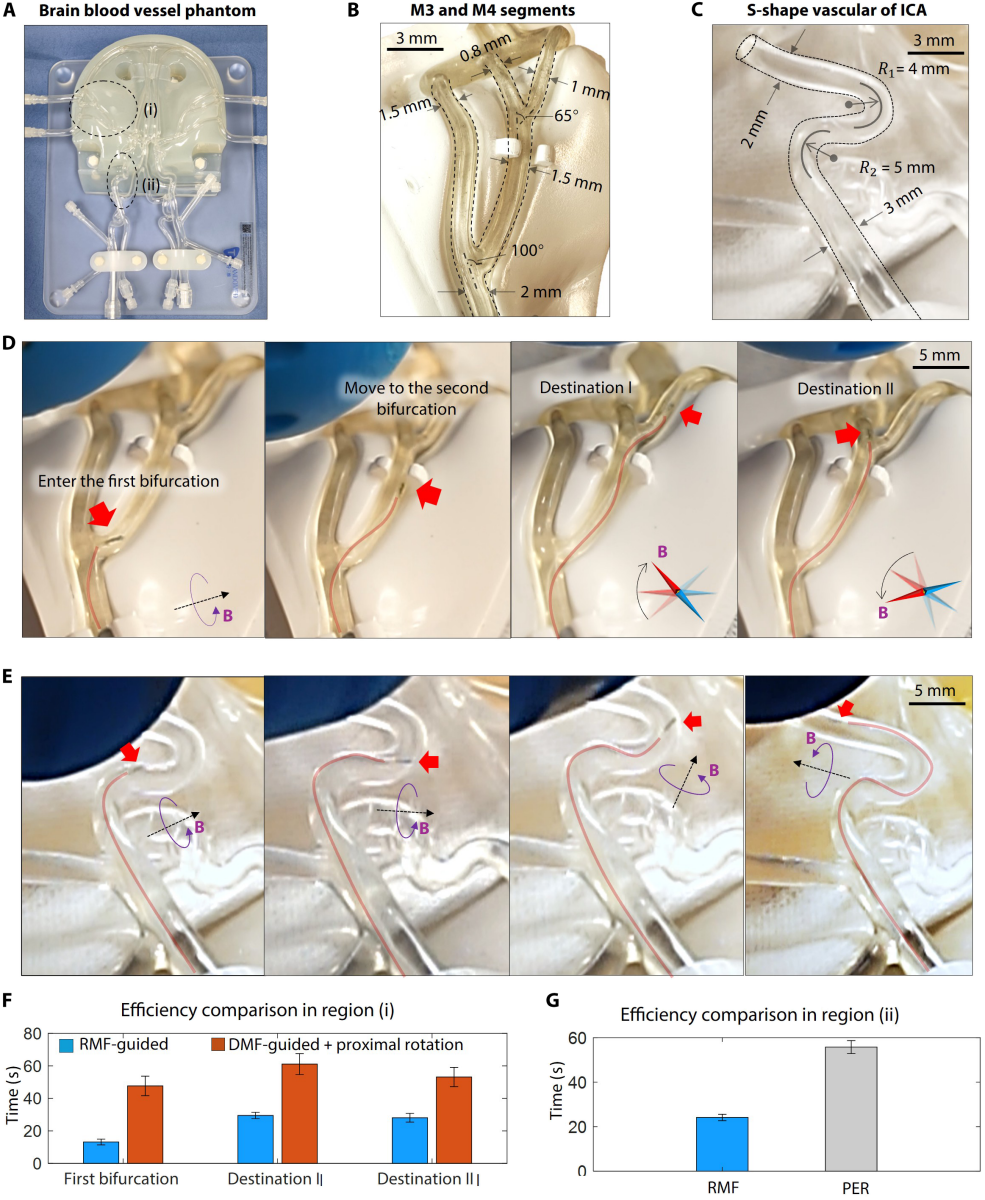
In vitro accessibility verification. (**A**) In vitro navigation in full-size human cerebral blood vessel model. Two regions of interest are as follows: (i) the M3 to M4 transition region of the MCA and (ii) the S-shape tortuous blood vessel of the ICA. (**B**) Dimension details of the phantom region (i). (**C**) Dimension details of the phantom region (ii). R1 and R2 are the curvature radius of the first and the second turn, respectively. (**D**) Keyframes of navigation in the region (i). The microcatheter navigated two bifurcations and arrived at two destinations in the distal branches. The red arrow highlights the position of the microcatheter tip. (**E**) Keyframes of navigation in region (ii). The microcatheter passes two continuous 180° turns with small radius curvature. Scale bar, 5 mm. (**F**) Navigation efficiency comparison of the region (i). Each group of tests is repeated 10 times. (**G**) Navigation efficiency comparison of the region (ii). Each group of tests is repeated 10 times.

As illustrated in Fig. 4A, two vascular segments have been selected for an accessibility demonstration, with detailed dimensions provided in Fig. 4 (B and C) and the access ports are provided in fig. S7B. Navigating the blood vessel bifurcations of the M3 and M4 segments [region (i)] requires first traversing a 3D tortuous bifurcated lumen, followed by accessing distal narrow branches with diameters of 1 and 0.8 mm. The experimental demonstration is depicted in Fig. 4D and movie S4. By applying the rotation-assisted steering strategy, navigation proves to be more efficient than using a static magnetic field across all bifurcations. A quantitative efficiency comparison for the region (i) between the RMF-guided and DMF-guided strategies [with proximal end rotation (PER) assistance] is shown in Fig. 4F. The results indicate that the RMF-guided approach is approximately three times faster in navigating the first bifurcation and about 1.9 and 1.7 times faster in reaching the first and second destinations, respectively. As the microcatheter advances, a robotic arm positions a rotatable magnet based on the microrobot’s location to provide the necessary magnetic guidance. The robotic arm–assisted permanent magnetic system is illustrated in fig. S2B.

The second demonstration was conducted in the 3D tortuous S-shaped segment of the internal carotid artery (ICA) within the silicone vascular model [region (ii)]. This segment features two continuous 180° turns with diameters ranging from 3 to 2 mm. When using conventional guidewires, careful navigation through this region is crucial to prevent damage to the blood vessel wall, which can occur due to increased friction from tight contact. In contrast, the rotation-assisted navigation strategy enhances navigation efficiency by reducing frictional forces. The experimental demonstration is illustrated in Fig. 4E and movie S5. The microcatheter was guided by a rotating magnetic field as it navigated through the S-shaped lumen. The direction of the RMF was adjusted to align with the shape of the phantom. This rotation-assisted strategy resulted in approximately twice the operational speed compared to the control group, where the microcatheter was passively inserted while its proximal end was manually rotated to facilitate navigation (Fig. 4G). Furthermore, we conducted a comparative study to evaluate the performance of the RMF-guided microcatheter against a conventional pre-bent guidewire (ZIPwire, Boston Scientific, OD: 0.89 mm), as illustrated in fig. S8A. The keyframes in fig. S8B demonstrate that, whereas the pre-bent guidewire navigates straight sections smoothly due to its hydrophobic coating, it encounters navigation challenges at the second turn of the S-shaped vascular phantom. Specifically, contact between the tip and the vessel wall leads to buckling as the contact area increases [fig. S8B(i to iv)]. Successful navigation with the guidewire necessitated repeated retraction, reinsertion, and proximal rotation, a time-consuming process with inherent clinical risks. In contrast, our rotation-assisted magnetic guidance system facilitated seamless navigation through the same phantom in a single attempt (fig. S8C), underscoring its superior effectiveness and efficiency. A detailed comparison video demonstration is shown in movie S5.

To enhance clinical validity, we conducted additional trials involving an experienced operator with specialized expertise in endovascular interventions (see fig. S7A). The same silicone model and magnetic field control strategy used in Fig. 4 (F and G) were used. The desired trajectory and insertion port are illustrated in fig. S7B. During navigation in region (i), the microcatheter was manually inserted and directed at the initial bifurcation to access the branch. Both DMF-guided and RMF-guided methods were evaluated in 10 trials. The results presented in fig. S7C demonstrate that experienced operators achieved navigation speeds 1.7 times faster with the RMF method, along with a 60% reduction in performance variability compared to the DMF method. For the navigation experiment conducted in region (ii), the operator manually advanced the microcatheter through the phantom using two distinct methods: RMF guidance and PER, while targeting the trajectory of the S-shaped segment, as detailed in fig. S7B. Operators using RMF exhibited navigation speeds 1.6 times faster and a 62% lower SD in stability when compared to those using PER, as shown in fig. S7D.

We further demonstrate the maintained steerability and rotational capability after long-distance vascular travel, as shown in fig. S9A. The phantom features three consecutive turns with varying degrees of curvature and diameters ranging from 3 to 0.5 mm. The total navigation distance covered was nearly 300 mm, with the most distal branch positioned at 165° relative to the entrance direction. A robotic arm–controlled permanent magnet is positioned beneath the phantom, and the desired magnetic field is calculated based on a preregistered trajectory. Depending on the degree of bending curvature, we alternate between directional magnetic actuation and rotation-assisted active bending. A detailed demonstration is presented in movie S6. A quantitative evaluation of the steerability after long-distance travel is provided in fig. S9 (B and C), showing that the proposed microcatheter offers advantages in navigation speed and the reachable workspace compared to the nonguided baseline.

### Treatment functions demonstration of MSRM

Previous studies have reported the use of rotational motion to mechanically break blood clots (*23*, *33*), but the aggressive mechanical motion of rigid rotors has raised safety concerns regarding blood vessel wall injury (*42*). Alternatively, researchers have demonstrated the use of thrombolytic drugs to accelerate blood clot clearance. However, systematic drug administration can lead to internal bleeding. The presented MSRM offers a hybrid solution by combining rotational physical interaction with local thrombolytic drug administration. As shown in Fig. 3H, the thrombolytic drug can be injected through the working channel and released at the openings on the rear end of the microrotor. The thrust flow carries the drug forward to the blood clot while switching the rotation direction induces backward flow to transport the clot debris to the supporting aspiration catheter. Herein, we use an in vitro thrombosis treatment as a case study to demonstrate the effectiveness of this hybrid strategy.

**Fig. 5.**
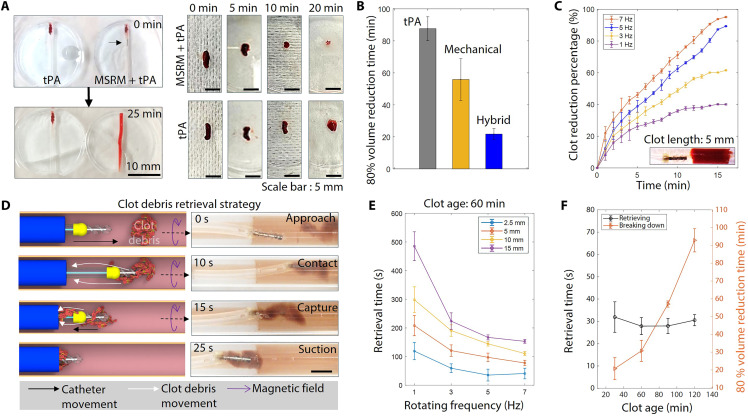
Endovascular treatment functions validation. (**A**) Experiment results to verify the blood clot–drug interaction enhancement. Clots are examined at intervals to assess volume reduction. (**B**) Treatment efficiency comparison using three methods to treat blood clots ( ϕ 2 mm × 5 mm, 30 min). Each group of tests is repeated three times. (**C**) Mechanical rubbing blood clot clearing efficiency at different speeds. The blood clot dimension: ϕ 2 mm × 5 mm. Each group of tests is repeated three times. (**D**) Schematic working principle and experimental demonstration of the blood clot retrieval strategy. (**E**) Blood clot retrieval efficiency on blood clots of different lengths. The retrieval time starts when the microcatheter exits and ends once all clot debris is collected. Each group of tests is repeated three times. (**F**) Blood clot retrieval efficiency and mechanical rubbing efficiency on blood clots of different ages. The retrieval time starts when the microcatheter exits and ends once all clot debris is collected. The rotating frequency is 5 Hz. Each group of tests is repeated three times.

As shown in fig. S6B, an artificial blood clot was fabricated in the phantom using a 37°C water bath. The microcatheter was navigated to the blood clot and rotational actuated to pump the thrombolytic drug, tissue plasminogen activator (tPA; 3 mg/ml, 0.2 ml, Actilyse, Boehringer Ingelheim), forward to the clot. A rotating permanent magnet below the phantom generated the required rotational magnetic actuation. Then, the microcatheter advanced and made contact with the blood clot. As clot removal progressed, the microcatheter was advanced to maintain contact for mechanical interaction. After 25 min, the clot was entirely cleared with no residual debris, which was approximately 10 times faster than in the control group. In the control group, the same amount of tPA was injected, and the phantom was kept in the water bath for the same time period as the experiment group. The detailed demonstration is presented in movie S7. To highlight the blood clot clearance performance, we performed another experiment and extracted the residual clots from both experimental and control groups at 0, 5, 10, and 20 min to visualize the volume reduction. The results are presented in Fig. 5A.

Further experiments were conducted to investigate the efficiency improvements of the proposed hybrid treatment (thrombolytic drug mixing and mechanical blood clot breakdown), as shown in Fig. 5B. A batch of blood clots (ϕ2 mm × 5 mm, cured for 30 min) was treated using three methods: thrombolytic drug treatment (tPA, 3 mg/ml, 0.2 ml, Actilyse, Boehringer Ingelheim), mechanical breakdown by the MSRM (rotating frequency: 5 Hz), and the proposed hybrid method. Notably, the proposed hybrid method demonstrated treatment speeds that were nearly 300 and 500% faster than the thrombolytic drug treatment and mechanical breakdown methods, respectively. In addition, we investigated the influence of rotating frequency on the clearance efficiency, as shown in Fig. 5C. Blood clots measuring 5 mm in length and 30 min in age were cleared by the proposed hybrid method under rotating frequencies of 1, 3, 5, and 7 Hz. For higher frequencies (5 and 7 Hz), the clot volume reduction rate was similar; they can achieve over 80% volume reduction in 15 min. The effects of tip stiffness and thrombolytic drug efficacy were further investigated, as shown in fig. S10. Both the soft (PDMS-based) and rigid (resin-based) tips were tested with and without the assistance of the thrombolytic drug. The comparison results suggested that the treatment performance was not compromised by the soft material design (97.36%).

The complexity of thrombus compositions makes in situ blood clot breakdown difficult (*43*). Fibrin-rich components are more resistant to shear forces and thrombolytic drugs. Red blood cell–rich clots are prone to fragmentation and can travel to distal branches, causing serious distal blockages. To address this, we proposed a retrieval strategy to collect unbreakable blood clot debris. By using both mechanical interaction and fluid manipulation, we can attract clot debris from small vascular structures to the aspiration catheter and capture it via suction. The detailed working principle is shown in Fig. 5D. Initially, the microcatheter rotates in a direction that generates a backward flow that attracts and captures the blood clot debris in the tip’s helical grooves. Then, the retraction of the microcatheter, combined with the flow, pushes the debris toward the rear end. Last, the debris is collected through negative pressure. The effectiveness of this strategy is experimentally verified, as shown in Fig. 5D and fig. S11. We use this method to capture two types of blood clots: red blood cell–rich and fibrin-rich clots. A detailed demonstration is presented in movie S8.

To identify the flow status of the retrieval mode, we use ultrasound imaging to monitor the clot retrieval process. As shown in fig. S12, artificial clot debris was placed in a silicone vascular phantom, and the MSRM was positioned close to the clot. Under a rotating magnetic field, the MSRM was actuated into retrieval mode, transporting the clot debris to the aspiration catheter [fig. S12(i and ii)]. The clot was then retrieved by the aspiration catheter through the combined forces of the rotating motion of the MSRM and negative pressure (104 Pa), as shown in fig. S12(iii). Furthermore, the Doppler signals [fig. S12(iv to vi)] indicated that the fluid in the lumen flowed toward the aspiration catheter, which is highlighted by green arrows. In contrast, the negative pressure generated by the aspiration catheter alone was insufficient to attract the blood clot, resulting in Doppler signals appearing only near the entrance of the aspiration catheter in the control group. Detailed demonstrations are provided in movie S9. We then characterize the effects of rotating frequency and clot volume (length) on retrieval efficiency. A batch of blood clots of varying lengths (2.5, 5, 10, and 15 mm) was retrieved while rotating at frequencies of 1, 3, 5, and 7 Hz. The results shown in Fig. 5E indicate that retrieval efficiency increases with rotating frequency and decreases with blood clot length. However, at higher frequencies, the efficiency levels off, and increased frequency may lead to greater invasiveness.

We also evaluated the effect of blood clot stiffness on retrieval and breakdown efficiency. Researchers have reported that the modulus of fabricated blood clots can be regulated by different curing times (clot age) (*44*). Blood clots measuring 5 mm in length of varying ages (30, 60, 90, and 120 min) were retrieved and broken down (rotating frequency: 5 Hz) using the proposed strategy. Our measurements indicate that the compression modulus of these blood clots is 23.7 ± 2.5, 25.7 ± 2.0, 31.2 ± 2.9, and 44.1 ± 3.9 kPa, respectively. The results illustrated in Fig. 5F suggest that the stiffness of the blood clot (age) influences the breakdown efficiency but cannot compromise the retrieval efficiency.

### In vivo demonstration

**Fig. 6.**
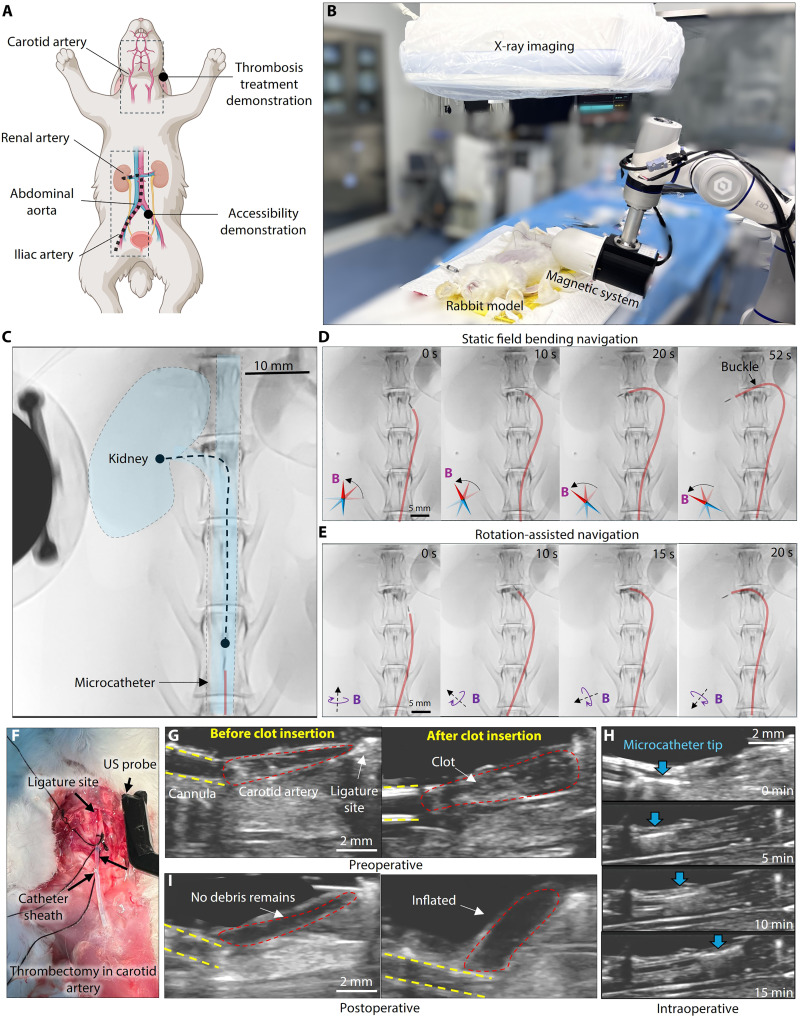
In vivo demonstrations in a rabbit model. (**A**) Two key regions: the carotid artery region and the iliac artery to kidney region. (**B**) System setup for x-ray imaging guided in vivo microcatheter navigation. (**C**) In vivo navigation to kidney artery. The dashed line highlights the desired trajectory and the bifurcation branches are highlighted in blue. Scale bar, 10 mm. (**D**) Navigation with static field actuation. The microcatheter can be steered toward the desired branch, but large friction at the tip may cause buckling on the microcatheter, as the arrow shows. (**E**) Navigation with rotational field guidance. The rotation-assisted steering mechanism can facilitate a smoother insertion process with a shorter duration and less tension on the catheter body. (**F**) System setup for in vivo blood clot clearance. (**G**) Preoperative stage of the in vivo blood clot clearance demonstration. The selected segment of the carotid artery is ligated at one end and connected to a catheter sheath at the other. In the preoperative stage, the blood clot is inserted through the sheath, filling the artery to the ligature site. (**H**) Intraoperative blood clot clearance demonstration. The microcatheter advanced deeper every 5 min. After 15 min, the blood clot is thoroughly broken down. (**I**) Postoperative stage of the in vivo blood clot clearance demonstration. The blood clot has disappeared from the imaging plane and cannot be observed after inflating the blood vessel.

Figure 6 (A and B) demonstrates the proposed navigation strategy and therapeutic functions in an in vivo rabbit model. In the navigation test, the microcatheter is inserted from the iliac artery and is aimed at navigating two turns with branch angles of ~30° and 90° to reach the renal artery (Fig. 6C). This navigation task is designed to simulate the conditions of navigating sharp angles in narrow human distal blood vessels with diameters ranging from 1 to 4 mm (*45*). X-ray imaging provides positional feedback, guiding the permanent magnetic system in generating the desired field for actuation. Both rotation-assisted active bending and static field bending are used to navigate the microcatheter, and the different bending curvatures are presented in Fig. 6 (D and E). Observations indicate that the rotation-assisted method mitigates microcatheter stress and prevents buckling that may occur with static field guidance. Furthermore, rotation-assisted navigation is ~50% faster than static field navigation. Movie S10 includes a detailed navigation and actuation process. Notably, the fluid delivery function is verified by injecting a contrast agent through the working channel, with rotational actuation enhancing the diffusion of the contrast agent.

To investigate the effectiveness of the MSRM’s drug-assisted mechanical breakdown function in vivo, we performed blood clot treatment within the rabbit carotid artery using ultrasound imaging to visualize the procedure, as shown in Fig. 6F. Initially, the distal end of the carotid artery was ligated and accessed with a catheter sheath. An artificial blood clot, created from the rabbit’s own blood, was inserted through the sheath and positioned inside the artery (Fig. 6G). The microcatheter was then inserted and navigated to the target. Similar to the in vitro experiments, tPA was injected at a dose of 3 mg/ml (0.2 ml), and the MSRM was actuated by a rotating magnetic field of 5 Hz. The catheter was advanced deeper every 5 min to ensure closer contact with the blood clot (Fig. 6H). After 15 min, the blood clot disappeared from the imaging plane. To confirm the thorough clearance of the clot, saline was injected to inflate the artery, providing additional details of the clearance, as shown in Fig. 6I. After confirming that the blood clot was broken down, we extracted the fluid from the artery, and no visible blood clot debris was found. The rate of blood clot clearance closely aligned with that of the in vitro experiments using the same dosage of the thrombolytic drug and the same magnetic field actuation, verifying the proposed microcatheter’s clinical application potential.

### Invasiveness evaluation in human placenta blood vessels

Previous studies have used micro device’s rotational motion for MT (*23*, *33*, *34*). Performance-enhancing designs, including sharp rigid profiles, high-friction coatings, and large rotational velocities, have been used to maximize blood clot clearance efficiency. However, these mechanical interactions raise safety concerns due to the fragile nature of blood vessels, particularly in narrow distal vessels with thinner walls and simpler support structures. In contrast, the proposed microcatheter adopts a soft material design and a tissue-friendly implementation method to balance efficacy and invasiveness. First, the rotatable helical structure is constructed from soft silicone (PDMS). We used a fabrication process that combines inverted molding and 3D printing to create a soft helical sheath that encases the permanent magnet, thereby avoiding hard contact with the inner vessel wall. This soft design does not compromise accessibility or therapeutic performance, as confirmed in earlier sections. Second, the proposed microcatheter’s ability to establish a fluid pathway for localized thrombolytic drug administration reduces the reliance on high rotational velocities. During implementation, the rotation frequency for rotation-assisted navigation is 2 to 5 Hz, and that for thrombectomy and drug delivery is 5 to 8 Hz. This frequency range, significantly lower than prior actuation methods (*23*), induces limited fluid stress on the blood vessel wall. A comprehensive comparison with the previously published rotor-tipped micromachine is provided in table S2. Among the current state-of-the-art technologies, the proposed microcatheter features the smallest OD, the lowest hardness, and the lowest angular velocity.

**Fig. 7.**
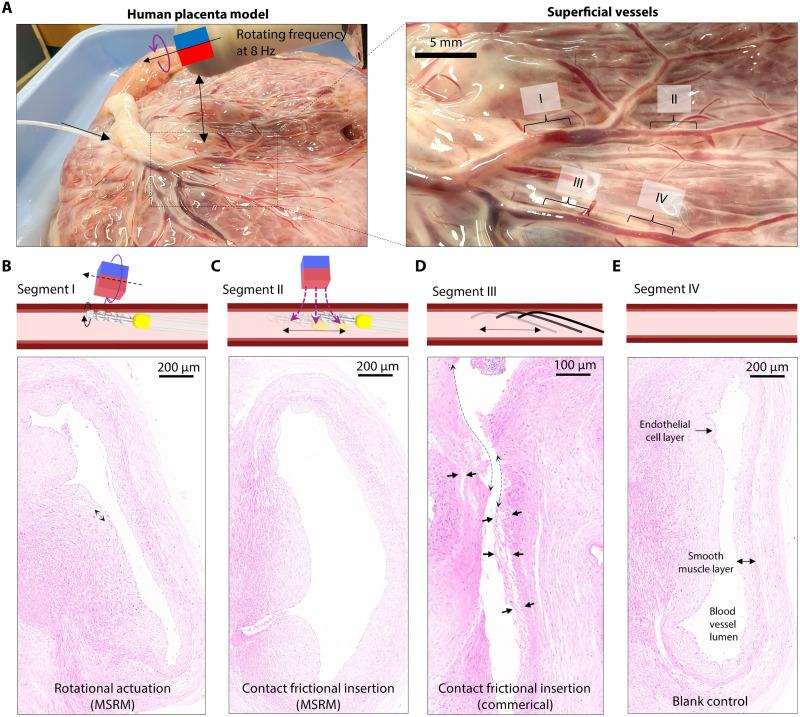
Invasiveness evaluation in human placenta blood vessels. (**A**) Setup of the invasiveness evaluation test. We choose four segments from the superficial blood vessels of the human placenta. In segment I, the proposed microcatheter contacts the blood vessel’s inner wall and is rotational actuated for 30 min. In segment II, the proposed microcatheter contacts the blood vessel’s inner wall and is repeatedly inserted 100 times. In segment III, a commercial guidewire is inserted into the blood vessel with tip contact on the vessel wall 100 times. Segment IV is blank control. (**B** to **E**) Example images of the histological analysis that show the physical impact on the endothelial cell layer and the inner vessel wall. The black arrows show the cell layer detachments, and the dashed line shows the torn and missing cell layer [rotational actuation and contact frictional insertion (commercial)].

To evaluate the potential impact of the microcatheter’s rotational actuation and contact frictional insertion on the blood vessel wall, we conducted an ex vivo invasiveness evaluation experiment using human placenta blood vessels, as illustrated in Fig. 7A. These arteries have diameters and wall thicknesses comparable to those of human cerebral arteries (*30*). We selected four segments of the superficial blood vessels, corresponding to four testing groups. In segment I, the microcatheter was inserted and rotated at 8 Hz in contact with the phantom’s inner surface for 30 min. In segment II, the microcatheter was frictionally inserted against the blood vessel’s inner wall 100 times. In segment III, a commercial guidewire (Boston Scientific, TRANSEND EX Guidewire) was repeatedly inserted into the blood vessel 100 times. Segment IV served as the blank control group. After the experiments, these four vessel segments were removed for damage assessment using hematoxylin and eosin (H&E) histological staining. The results, presented in Fig. 7 (B to E), indicate that the blank control group displayed an intact inner blood vessel wall, whereas the contact friction insertion (commercial) group exhibited notable damage to the inner cell layers, including detachments and missing layers. In contrast, the rotational actuation and contact frictional insertion (MSRM) groups showed minimal missing cells, with no detachment observed.

## DISCUSSION

In this study, we introduce a submillimeter magnetically actuated catheter designed to access narrow, tortuous vessels and perform endovascular treatments. The proposed microcatheter offers three notable advantages over previously published magnetic microcatheters. First, its miniaturized design and rotation-assisted navigation strategy ensure superior accessibility in narrow distal blood vessels (≤2 mm). Second, our microcatheter integrates multiple functionalities, including active steering, rotation-assisted navigation, accelerated drug-thrombus interactions, mechanical breakdown, and debris retrieval. This all-in-one design enhances treatment efficiency and offers a potential method for physically retrieving drug-resistant components from blood clots. Last, the soft tip design and tissue-friendly actuation strategy help mitigate the risk of vascular injury during navigation and actuation, as demonstrated in previous sections. A detailed comparison with the state of the art is provided in table S3.

Researchers have proposed the helical propulsion mechanism that converts rotational torque into axial motion to improve pushability (*30*). Alternatively, our work used wireless magnetic actuation to rotate the tip and dynamically modulate frictional interactions, preventing buckling and looping. This design proactively reduces frictional resistance and avoids singular configurations, prioritizing smooth interactions to minimize vascular stress rather than relying on friction for advancement. Moreover, MSRM wirelessly controls both rotation and steering through a unified external magnetic field, simplifying control integration. Furthermore, the proposed microcatheter decouples the rotational and advancing motions, which enhances functional versatility, including improved clot-drug interactions, mechanical clot disruption, and debris retrieval.

Classical mechanics approaches can predict the bending behavior of soft continuum robots, improving motion and force control and predicting shape and stress conditions (*46*–*48*). However, applying these techniques to the proposed microcatheter presents challenges. Characterizing the catheter body’s material properties is difficult due to nonlinear behavior and heterogeneity, compromising accuracy. Furthermore, fabrication errors with the embedded permanent magnet can lead to inconsistencies in actuation parameters, whereas manual assembly may introduce additional discrepancies across different devices. Alternatively, this work used a lookup table (LUT) that relates the actuation magnetic field to the steering angle, offering a practical and efficient solution for desired magnetic field calculation. Similar methods are used by researchers for building control framework for continuum robots that have complex structure design or high nonlinearities (*49*, *50*).

Incorporating sensing modules into microcatheter systems could enhance control accuracy and provide essential information regarding the robot’s shape, orientation, and stress conditions. One effective approach involves embedding internal sensing elements such as Hall-effect sensors, microcoils, and fiber Bragg gratings (FBGs) within the catheter (*31*, *51*, *52*). Integrating these sensing modules at submillimeter scales poses several challenges related to fabrication and actuation. For example, incorporating multiple optical fibers required for FBGs, each with a total diameter of ~250 μm, into a catheter with an overall diameter of less than 1 mm while also leaving sufficient space for a working channel is a complex task (*25*). To optimize spatial constraints, researchers have developed a triplet design that accommodates three optical fibers within a flexible needle with an OD of 0.6 mm (*53*). Although this design could be beneficial for our microcatheter, the increased stiffness from the optical fibers restricts the catheter’s bending range and increases the demands on the external magnetic actuation field (*54*, *55*). Alternatively, the second approach is to include external sensing modules, which use various clinical imaging techniques for effective real-time monitoring. This kind of method will not occupy the space inside the microrobot and could provide real-time accurate position feedback. Fluorescence imaging, despite its associated radiation exposure risks, remains the gold standard and is used by many researchers to navigate microcatheters (*15*, *30*, *56*). Magnetic resonance imaging (MRI) presents a radiation-free option that offers high-contrast visualization of soft tissues, whereas ultrasound provides a portable, high-resolution imaging solution devoid of radiation, thereby facilitating safer intervention strategies (*57*, *58*).

The length of the rigid tip on a microcatheter substantially affects its navigation within brain vessels, particularly at bifurcations. During steering, the microcatheter must conform to the vessel shape, which may lead to reversible deformation or straightening of the vessel. The use of rigid distal tips (RDT) raises the risk of damaging the vessel, emphasizing the need for a design that avoids contact with the vessel wall when transitioning from trunk arteries to cortical branches. Key parameters influencing accessibility at bifurcations include trunk vessel diameter ( $D_{\text{trunk}}$ ), branch vessel diameter ( $D_{\text{branch}}$ ), and the branch angle ( $\varphi_{\text{branch}}$ ), as depicted in fig. S13, where the yellow bounding box illustrates the effective area of the distal rigid tip. To assess the accessibility of the current microcatheter design and two proposed downsized alternatives, we analyzed anatomical vessel data from the MCA (table S4) (*59*). The current design provides full access to specific bifurcations, such as the intermediate trunk–angular artery and the inferior trunk–temporo-occipital/angular arteries while offering partial access at most others. Downsizing the device could enhance overall accessibility for navigating all targeted bifurcations larger than 0.8 mm. The existing no-contact rule, which is based on rigid tip diameter, does not consider the soft helical structure’s deformability, potentially underestimate the navigation capacity. In addition, the proposed rigid tip length aligns with state-of-the-art microcatheter designs that have been validated for potential clinical applications (table S5), confirming its suitability for clinical applications.

Although friction can occur along any part of the microcatheter, the proposed design specifically addresses accessibility challenges caused by friction between the distal tip and vessel wall when navigating lateral branches or sharp turns. Insertion difficulties due to body-vessel wall contact could be mitigated through a stiff gradient design (to maintain pushability) and low-friction material coatings on the catheter body (*25*, *60*). The proposed microcatheter features a stiffness gradient along its body, achieved by connecting tubes with varying elastic modulus, with the proximal end being stiffer and the distal end softer. Experimental evaluation of the influence of the catheter body-wall contact was conducted using a 3D-printed cerebral vascular phantom. This model replicates physiological features such as S-shaped turns, bifurcations, and transitions from large-diameter trunk arteries to narrow branches, with exact dimensions provided in fig. S14A. With the same setup described in fig. S1A, the microcatheter passively advanced through the first turn but encountered resistance at the second turn due to tip-vessel friction. Applying rotational magnetic actuation mitigated this friction, enabling redirection [fig. S14B(iv to vi)]. At the bifurcation, alternating static (steering) and rotational (friction reduction) magnetic fields allowed navigation into three target branches even with multiple catheter body-vessel wall contacts [highlighted by the green arrow in fig. S14B(vi)]. Detailed results are shown in movie S11.

In realistic scenarios, the operational space is often limited, which can lead to tension accumulation manifesting as buckling or looping, different from what we observed in Fig. 2A. An experimental demonstration to clarify the tension and shape status of the microcatheter when navigation in the vascular system is presented in fig. S15. With the same experiment settings as figs. S2A and S7B(i), the microcatheter was manually advanced and magnetically guided by both DMF and RMF to the bifurcated branch. Under the DMF guidance, steering into the branch converted part of the insertion force into normal force on the vessel wall, increasing tip friction. Further advancement causes proximal buckle [close-up view, fig. S15A(ii and iii)]. Applying a rotating magnetic field reduced tip friction through rotational motion, enabling successful branch insertion [fig. S15B(i to iii)]. The successful navigation of narrow bifurcations demonstrates the efficacy of the rotation-assisted steering strategy in confined endovascular environments. Detailed video demonstration is detailed in movie S12.

The proposed retrieval procedure follows the procedure of mechanical interactions (e.g., device-clot engagement) and thrombolytic drug administration (e.g., clot dissolution). During these stages, fragile clot components are fragmented by mechanical forces, dissolved by pharmacological agents, and subsequently collected via the aspiration catheter. However, red blood cell–rich clots remain prone to fragmentation during device retraction, often necessitating slow, controlled retraction to ensure complete clot removal. Clinically, preprocedure imaging (e.g., CT/MRI) is critical to evaluate clot composition (friable versus organized) and optimize the retrieval strategy (*61*). To further mitigate fragmentation risks, balloon-guided catheters (for flow arrest) and aspiration catheters (for fragment capture) are used synergistically (*62*, *63*).

Porcine blood clots were selected for in vitro experiments due to their established use in evaluating microrobot systems (*19*, *64*–*65*), offering a baseline model to evaluate the effectiveness of the blood clot treatment. Although porcine plasminogen exhibits reduced tPA sensitivity, this highlights the mechanical interaction’s importance in the system. As shown in Fig. 5J, clots treated with tPA and mechanical interaction were around three times faster than pure tPA administration. This synergy aligns with a prior work (*65*), where thrombolytic agents combined with mechanical interaction drive rapid clearance. For the in vivo blood clot treatment, rabbit blood is used instead of porcine blood to avoid immune reactions, which could confound physiological outcomes. It follows the protocol of previous works (*66*–*69*).

Ex vivo and in vivo tests demonstrated the effectiveness and efficiency of the thrombolytic drug-assisted mechanical thrombectomy. Notably, the proposed method can not only rapidly increase the local drug concentration with a small dosage but also accelerate the clearance process through mechanical interaction. Systematic experiments validate that the dosage and treatment duration are theoretically superior to traditional thrombolytic drug administration and catheter directed thrombosis treatments. In future work, we plan to quantitatively evaluate the optimal drug dosage and magnetic actuation parameters through animal experiments.

## MATERIALS AND METHODS

### Magnetic field actuation method

The magnetic field control framework, illustrated in fig. S16, consists of two phases: preoperative and intraoperative. In the preoperative phase, the microcatheter’s trajectory is first established using imaging modalities [in vitro: camera; in vivo: DSA (digital subtraction angiography)]. The desired magnetic field at the via points is then calculated, taking into account the microcatheter’s trajectory and potential interference with the imaging modalities. Subsequently, the position and orientation of the actuation magnet are determined to achieve the desired magnetic field at these via points, followed by planning the robotic arm’s trajectory to align with the actuation magnet’s path. During the intraoperative phase, the microrobot is manually inserted into the blood vessel, using real-time DSA imaging for positional feedback. The robotic arm’s configuration is adjusted based on the microcatheter’s position and the preoperative calculations, enabling accurate navigation along the planned trajectory throughout the procedure.

In the preoperative process, we first establish the trajectories for both the microcatheter and the actuation magnet. The trajectory of the microcatheter ( $T_{r}$ ) is obtained either from the CAD model (in vitro) or DSA imaging (in vivo). We then decompose $T_{r}$ into specific via points ( $P_{v}^{n}$ , where the superscript *n* indicates the *n*th point) and add a constant relative distance to each via point. This allows us to derive the trajectory for the actuation magnet ( $T_{a}$ ) as follows

$$T_{a}=\left[P_{a}^{1},P_{a}^{2}\ldots P_{a}^{N}\right]=\left[P_{v}^{1}+P_{r},P_{v}^{2}+P_{r},\ldots P_{v}^{N}+P_{r}\right]$$ (1)

where $P_{r}$ is the relative position N is the total number of via points, and $P_{a}^{n}$ is the position of the actuation magnet corresponding to the *n*th via point $P_{v}^{n}$ . The actuation magnet is designed to move in sync with the microrobot along a predetermined fixed trajectory. This preplanned trajectory helps avoid interference with other medical devices and maintains the quality of imaging.

Second, we derive the desired magnetic field at the via points and identify the direction of the magnetic moment for the external actuation magnet to apply the desired magnetic field at the robot’s position.

Static field bending mode: The bifurcations are first identified and the bending angle on the *i*th bifurcation can be calculated using the following formula

$$\theta_{d}^{i}={\text{cos}}^{-1}\left(D_{v}^{k_{1}}\cdot D_{v}^{k_{2}}\right)$$ (2)

where $D_{v}^{n}$ is the unit direction vector of the *n*th via point, and $k_{1}$ and $k_{2}$ denotes the points where the bifurcation starts and the steering process ends. $\theta_{d}^{i}$ , $k_{1}\leq i\leq k_{2}$ is the bending angle. The desired magnetic field can be obtained by the bending angle using the LUT method

$$B_{d}^{i}=M\left(\theta_{d}^{i}\right)$$ (3)

where $B_{d}^{i}$ is the desired magnetic field on the *i*th point. M represents the searching in the LUT, which is obtained by experiments offline.

Static field navigation mode: To enhance the insertion process of the microcatheter within vascular systems, a magnetic field perpendicular to the tangent direction is applied to maintain the microcatheter tip’s orientation. For ease of implementation, the required magnetic field is defined by the cross product of the tangent direction vector and the axis vector

$$B_{d}^{j}=B{\hat{B}}_{d}^{j}=B\cdot \left\{ \begin{array}{c} D_{v}^{j}\times {\left[0\;0\;1\right]}^{T},{\hat{B}}_{d}^{j}\neq \left[0\;0\;1\right]\\ {\left[0\;1\;0\right]}^{T},{\hat{B}}_{d}^{j}=\left[0\;0\;1\right] \end{array}\right.$$ (4)

where j represents the index of the via points that are not included in the steering process. B denotes the strength of the magnetic field, which is determined by the operator. The magnetic field produced by the spherical permanent magnet used in this work (Ф 50 mm, N52) can be described using the dipole model as follows

$$B_{a}=\frac{\mu_{o}}{4\pi }\left[\frac{3\hat{P_{r}}\left(\hat{P_{r}}\cdot m\right)-m}{{|P_{r}|}^{3}}\right]$$ (5)

where $B_{a}$ is the magnetic field applied on the microrobot and $\mu_{o}$ is the permeability constant, $\hat{P_{r}}$ is the unit vector of $P_{r}$ , and m is the magnetic moment and can be expressed as

$$m=M\cdot \hat{m}=M\cdot \left[{\hat{m}}_{x},{\hat{m}}_{y},{\hat{m}}_{z}\right]$$ (6)

M is the magnitude of the magnetic moment of the actuation magnet. With the given $P_{r}$ , we can calculate the optimized $\left[{\hat{m}}_{x},{\hat{m}}_{y},{\hat{m}}_{z}\right]$ by minimizing the difference between the desired magnetic field $B_{d}^{n}$ and the magnetic field generated by the external magnet

$$\begin{array}{c} \underset{{\hat{m}}_{x},{\hat{m}}_{y},{\hat{m}}_{z}}{\text{min}}|B_{d}^{n}-B_{a}\left({\hat{m}}_{x},{\hat{m}}_{y},{\hat{m}}_{z}\right)| \end{array}$$ (7)

Rotating field: The desired magnetic field of the *n*th via points can be described as

$$B_{d}^{n}\left(t\right)=B_{0}^{n}\cdot \text{cos}\left(\mathrm{wt}\right)+\left(D_{v}^{n}\times B_{0}^{n}\right)\cdot \text{sin}\left(\mathrm{wt}\right)$$ (8)

where $B_{0}^{n}$ refers to the initial vector established by the cross product of the direction vector $D_{v}^{n}$ and a coordinate axis, with its magnitude predetermined by the operator. w represents the angular velocity, and t denotes time.

The step motor installed on the robotic arm enables rapid rotational motion. We define the rotation axis as $u=\left[u_{x},u_{y},u_{z}\right]$ . The dipole moment rotates around this axis, which is perpendicular to the magnetization. The direction of the dipole moment after rotation by an angle wt can be calculated as follows

$$\hat{m}\left(u,\mathrm{wt}\right)={\hat{m}}_{0}\cdot \text{cos}\left(\mathrm{wt}\right)+u\times {\hat{m}}_{0}\cdot \text{cos}\left(\mathrm{wt}\right)$$ (9)

Substituting to $\hat{m}$ to the dipole model, we can calculate the magnetic field at time *t* as

$$\begin{array}{c} B_{a}\left(u,t\right)=\frac{\mu_{o}}{4\pi }\left[\frac{3\hat{P_{r}}\left(\hat{P_{r}}\cdot M\hat{m}\left(u,\mathrm{wt}\right)\right)-M\hat{m}\left(u,\mathrm{wt}\right)}{{|P_{r}|}^{3}}\right] \end{array}$$ (10)

The optimal rotation axis can be obtained through solving the optimization problem below

$$\underset{u_{x},u_{y},u_{z}}{\text{min}}\sum_{t=0}^{T}|B_{d}^{n}\left(t\right)-B_{a}\left(u,t\right)|$$ (11)

### Magnetic system configuration

For different experiments, the robotic arm–based permanent magnet system adopts varied configurations. For in vitro experiments described in Figs. 2A and 5 (A and D) and fig. S14, the actuation magnet was positioned below the phantom (fig. S1A) and the robotic arm synchronizes its motion with the microcatheter tip (tracked via camera feedback) to maintain a fixed vertical alignment. Field strength is controlled by adjusting the magnet-to-microcatheter distance, whereas the magnetic field direction is controlled via robotic arm alignment of the magnet’s magnetic moment. A stepper motor spins the magnet to generate a rotating field perpendicular to its magnetization axis ( $x^{e}$ ), enabling precise control of actuation parameters. For in vitro and in vivo experiments described in Figs. 4 and 7 and fig. S15, the magnet moves at pace with the microcatheter above the phantom. The DMF is generated by controlling the actuation magnet’s orientation and relative position. The RMF is generated by controlling the actuation magnet’s rotation axis’ orientation, rotation speed, and relative position. Specifically, for in vivo experiments, the system was positioned beside the testing model, generating a 5- to 10-Hz rotating field (10 mT) and static directional field (75 mm safe distance) for fluoroscopy-compatible navigation. Hardware specifications are detailed in table S6.

### Fabrication of the PDMS-based helical catheter tip

In this work, we first create a soft helical sheath using molding techniques and attach it to the outer surface of a micro permanent magnet with a waterproof adhesive treatment. Next, we combine the helical sheath with the 3D-printed miniaturized ball joint and the working channel, creating a soft, rotatable catheter tip. Specifically, the fabrication process begins with the creation of negative molds for the helical tip using 3D printing (NanoArch S130, BMF Precision, China). These molds undergo ultraviolet light curing at 100°C for 60 min, followed by 15 min of plasma treatment and 3 hours of silane steam treatment using pure triethoxy(1*H*,1*H*,2*H*,2*H*-perfluoro-1-octyl)silane. This process renders the resin mold hydrophobic, aiding later demolding. The molds are held together with nonmagnetic clips and submerged in thoroughly mixed PDMS fluid (base and cross-linker mass ratio of 10:1). A permanent magnet (NdFeB, φ0.3 mm x 2 mm) is inserted into the mold before curing at 70°C for 3 hours. After demolding and manual removal of the base material, the PDMS-based helical tip is obtained. The ball joint chamber and hollow ball joint are 3D-printed using the same resin as the negative molds. The microcatheter is then assembled and prepared for testing. Detailed fabrication steps can be found in fig. S17. The prototype discussed here measures 800 μm in diameter, but with the use of the advanced microassembly platform (*70*), we can further reduce its size to ~100 μm.

### Physical model specifications

The physical models used in this work were fabricated using various methods and materials. The models for demonstrating rotation-assisted active steering (Fig. 2, A and B) were 3D printed using polylactic acid (PLA) (Bambu Lab P1S). For insertion force comparisons (Fig. 2C), we self-made the model using PDMS. The silicone phantom simulating human brain blood vessels (Fig. 4, A to C) was purchased from Ningbo Trando 3D Medical Technology Co. Ltd. The inner vessel diameters were obtained directly from the vendor’s CAD models, which are based on clinical CT and MRI imaging data to ensure anatomical accuracy. In addition, the model for assessing bending and rotation (fig. S4) was self-made using PLA (Bambu Lab P1S), whereas the navigation test model (fig. S9) was self-made using PDMS. For testing the maintained accessibility after continuous turns (fig. S14), we customized a 3D-printed model using clear resin (UnionTech).

### MSRM and blood clot contact simulation

The microcatheter is rotated at 5 Hz and inserted into a blood clot measuring 1.5 mm in diameter and 3 mm in length. The blood clot has a modulus of 957 Pa and a fraction shear force of 499 Pa, based on clots made from human platelet–rich plasma (PRP) blood samples. The maximum insertion depth is 0.3 mm, corresponding to the length of the tip.

### Preparation of the artificial blood clot

In the in vitro blood clot clearance test, the artificial blood clot is made by mixing the sterile pig blood anticoagulated with sodium citrate (Guangzhou Hongquan Biotechnology Co. Ltd.) with calcium chloride (0.5 mol/ml, 10 μl) fluid and curing the mixture at 36.5°C. In the in vitro test, the curing time is 30 min. In the clot debris retrieval test, the fibrin-rich blood clot is first cured for 1 hour and washed out the extra red blood cells. The red blood cell–rich blood clot is cured for 30 min. In the in vivo thrombectomy test, the blood clot is made of the rabbit’s blood and cured for 30 min.

### Remote microcatheter insertion method

We use the design of the filament extruder in the material extrusion–based 3D printers to realize insertion motion control. The working principle is described in fig. S18. The insertion machine uses torque and a pitch system to feed and retract the microcatheter in precise amounts. The microcatheter working channel has stepped stiffness for sufficient pushability at the proximal and flexibility at the distal end. We experimentally verified the insertion performance through magnetic navigation under x-ray imaging. The setup is presented in fig. S19A. The host computer remotely controls the robotic arm, the step motor of the magnet, and the catheter advance machine. The magnetic source is positioned at one side of the phantom, leaving enough imaging space as shown in fig. S19B. The contrast agent (120 mg/ml, sodium diatrizoate hydrate fluid) was first injected into the phantom to outline the vascular model under x-ray. As the embedded permanent magnet is radio-opaque, it acted as an x-ray marker for the localization of the catheter tip. The robotic arm adjusted the position and orientation of the permanent magnet to steer the microcatheter to the desired branch, where the desired bending angle and external magnetic source pose were precalculated. Some keyframes during the navigation into the branches are depicted in fig. S19C.

### Rabbit model preparation

Two adult New Zealand rabbits used in the experiment were food restricted for 24 hours before the procedures. The ethical approval from the Institutional Animal Care and Use Committee was obtained before the research (AMS D2303010R). Before the intervention, the rabbit was anesthetized by injecting Zoletil 50 (5 mg/kg) into the ear vein. For maintenance during the procedure, half of the initial dose was given intravenously. Before inserting the catheter, the ear vein was located and treated with a 2% lidocaine gel to reduce pain. A small catheter was carefully inserted into the vein using a tourniquet to improve visibility of the vein, and a saline flush was performed to ensure the catheter was clear and functioning properly. Navigation test: Once the rabbit’s vital signs were stable, it was placed on its back on a temperature-controlled surgical table, and its limbs were secured to keep it still. The surgery continued with an incision in the inner thigh, exposing the femoral artery, and inserting a 5F sheath. Blood clot clearance test: After stabilizing the animal under anesthesia, the rabbit was placed in a supine position with its limbs restrained using ropes and secured to the operating table. The fur on the neck was shaved, and the surgical site was disinfected with iodine, followed by alcohol deiodination. A longitudinal skin incision (~2 cm in length) was made using a scalpel. Subcutaneous fascia and muscle layers were dissected to expose the carotid artery. The artery was carefully separated using forceps, and a 5F vascular access sheath was inserted for cannulation.

### Human placenta model preparation

The human placenta was collected from the Prince of Wales Hospital, which was approved and overseen by the Joint Chinese University of Hong Kong (CUHK)–New Territories East Cluster Clinical Research Ethics Committee (ref. no. 2020.384). The placenta used in this study was donated by a pregnant woman who underwent a cesarean section at Prince of Wales Hospital in collaboration with the Department of Obstetrics and Gynaecology at CUHK. The patient provided written informed consent to participate in the study. To be eligible for the study, pregnant women had to be healthy, between the ages of 20 and 45, of any ethnic origin, and give birth via cesarean section between 37 and 42 weeks of gestation. They also needed to have a singleton pregnancy, be determined as healthy by their treating physicians based on laboratory results, physical examination, and medical history, and have the ability to provide written informed consent voluntarily. Participants who had abnormal prenatal development, hypercholesterolemia, a family history of stroke or vascular diseases, diabetes, gestational diabetes, or cancer were excluded from the study. Once collected, the placenta was thoroughly washed with saline to remove any impurities, and the blood inside the blood vessels was carefully drained to prevent blockage.

## Supplementary Materials

### The PDF file includes:

Supplementary Text Figs. S1 to S19 Tables S1 to S6 Legends for movies S1 to S12 References
