# Supplementary material for: A magnetically actuated microcatheter with soft rotatable tip for enhanced endovascular access and treatment efficiency
Source: Sci Adv. 2025 Jun 20;11(25):eadv1682. doi: 10.1126/sciadv.adv1682 (PMC13108821; doi:10.1126/sciadv.adv1682)
Supplement: Supplementary file 1 — Supplementary Text Figs. S1 to S19 Tables S1 to S6 Legends for movies S1 to S12 References [file sciadv.adv1682_sm.pdf]

Science Advances
AAAS

Supplementary Materials for

A magnetically actuated microcatheter with soft rotatable tip for enhanced endovascular access and treatment efficiency

Moqiu Zhang *et al.*

Corresponding author: Lidong Yang, lidong.yang@polyu.edu.hk; Bonaventure Yiu Ming Ip, bonaventureip@cuhk.edu.hk; Thomas Wai Hong Leung, drtleung@cuhk.edu.hk; Li Zhang, lizhang@cuhk.edu.hk

Sci. Adv. **11**, eadv1682 (2025)
DOI: 10.1126/sciadv.adv1682

The PDF file includes:

Supplementary Text
Figs. S1 to S19
Tables S1 to S6
Legends for movies S1 to S12
References

Other Supplementary Material for this manuscript includes the following:

Movies S1 to S12

Supplementary Text

Supplementary Figures

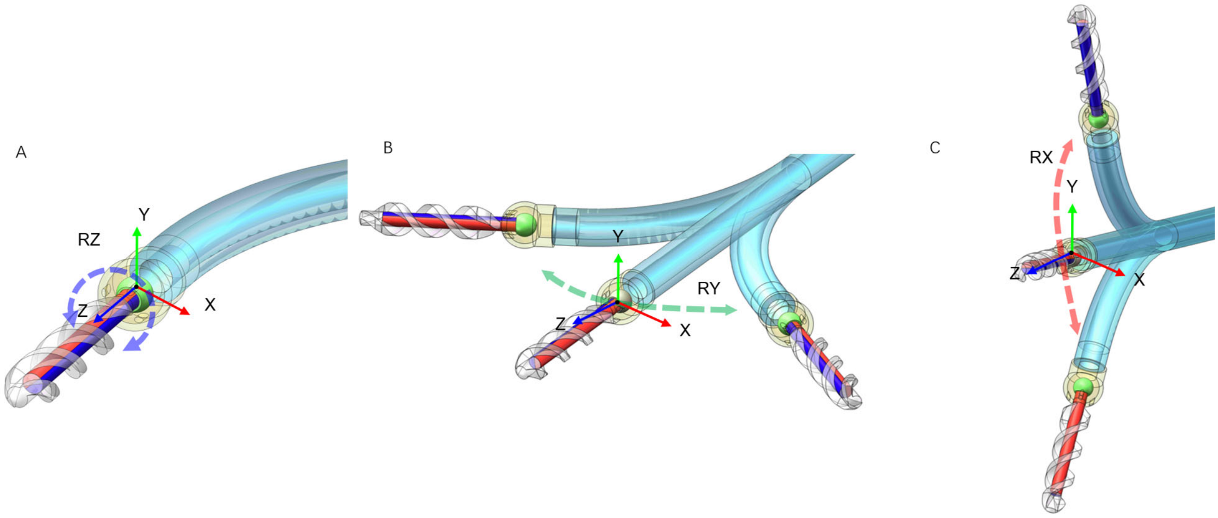

Fig. S1 Schematic drawing of the 3D rotational motion of the microcatheter. (A) Rotation around the Z axis (RZ). (B) The magnetic moment of the embedded permanent magnet aligns with the X axis and can then rotate around the Y axis (RY). (C) The magnetic moment of the embedded permanent magnet aligns with the Y axis and can then rotate around the X axis (RX).

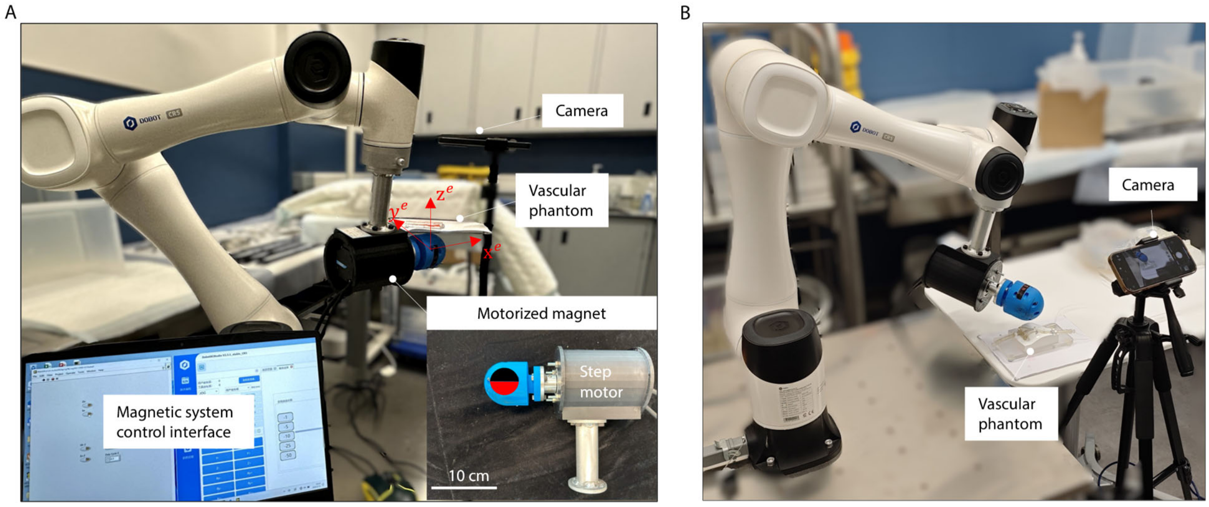

Fig. S2. Robotic arm-assisted magnetic system's configurations. (A) Experiment setup. A 5-DOF robotic arm holds a motorized spherical permanent magnet, with the magnetization displayed in the close-up view on the bottom right. (B) Experiment setup for navigation tests in full-size human brain vascular phantom.

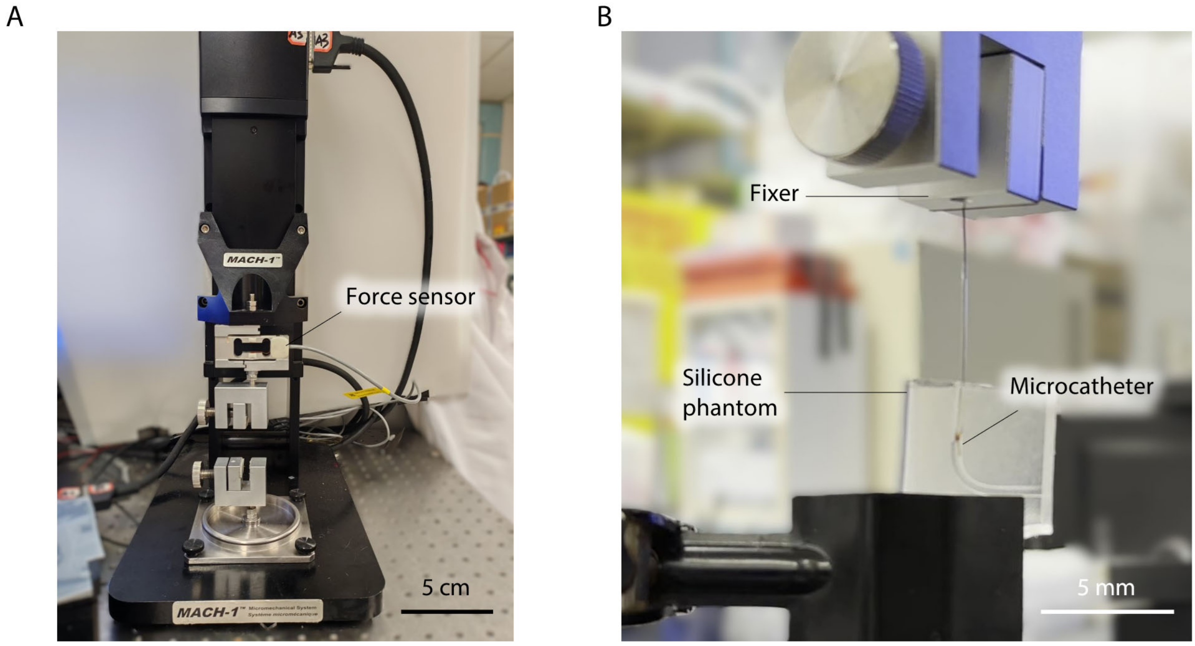

Fig. S3. Setup of the insertion force experiments. (A) MACH-1 force testing platform (B) Close-up view of the insertion fixer, the catheter and the silicone phantom. The sensor is purchased from a local vendor (Arizon, model: AR8101M, Range: 10N, Resolution: 0.1%, Single axis).

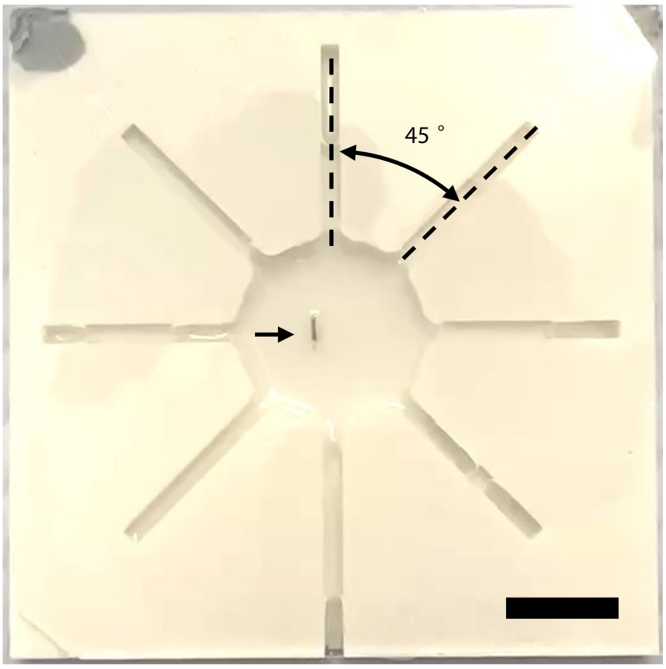

Fig. S4. The bifurcation phantom for bending and rotating assessment. The scale bar is 5 mm.

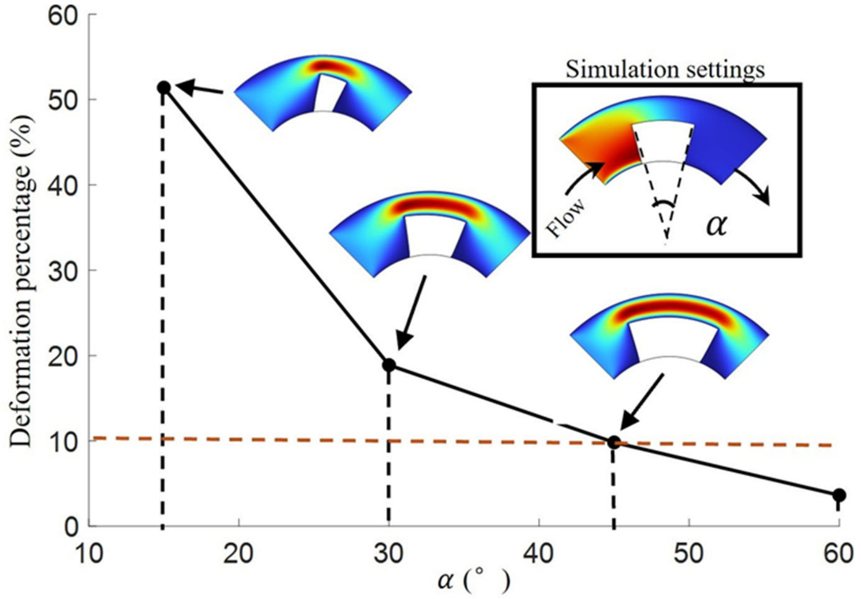

Fig. S5. Soft fin deformation under rotating field. The fins were exposed to continuous blood flow with a maximum flow rate of 1.1 cm/s, which was the flow rate under the desired rotation frequency of 8 Hz. The viscosity of the fluid was set to 20 cP to represent the blood viscosity at stagnant condition. We select α= 45˚ to have a deformation less than 10%. The deformation is calculated by the area difference divided by the original area.

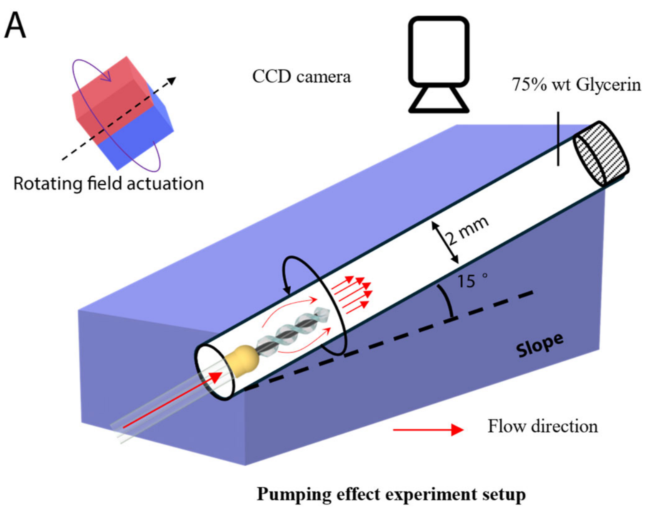

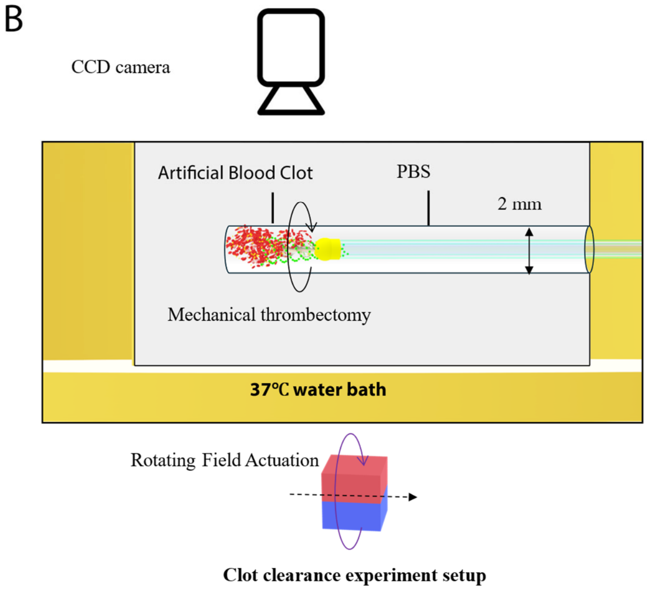

Fig. S6. Experiment settings of the pumping effect tests and in vitro blood clot clearance tests. (A). The system design for the pumping effect demonstration, which was comprised of a plastic lumen that was tilted at 15° on a slope and filled with a 75% glycerin solution. To initiate the demonstration, 15 µL of red dye was injected from the working channel and released at the opening located on the distal end of the MSDM. The external magnetic field actuates the helical tip to rotate and propel the dye uphill. The red dye boundary's position indicates the fluid mixing speed and is extracted from the CCD camera images. (B). System setup for blood clot mechanical rubbing test. The system consists of a silicone lumen, which is immersed in a 37°C water bath, and a microcatheter that is magnetically actuated by a rotating magnetic field. The microcatheter mechanically rubs against an artificial blood clot, which is fabricated inside the silicone lumen that is filled with PBS. From the images captured by the CCD camera, we derive the area of the red clot, which serves as an indicator of the clear rate.

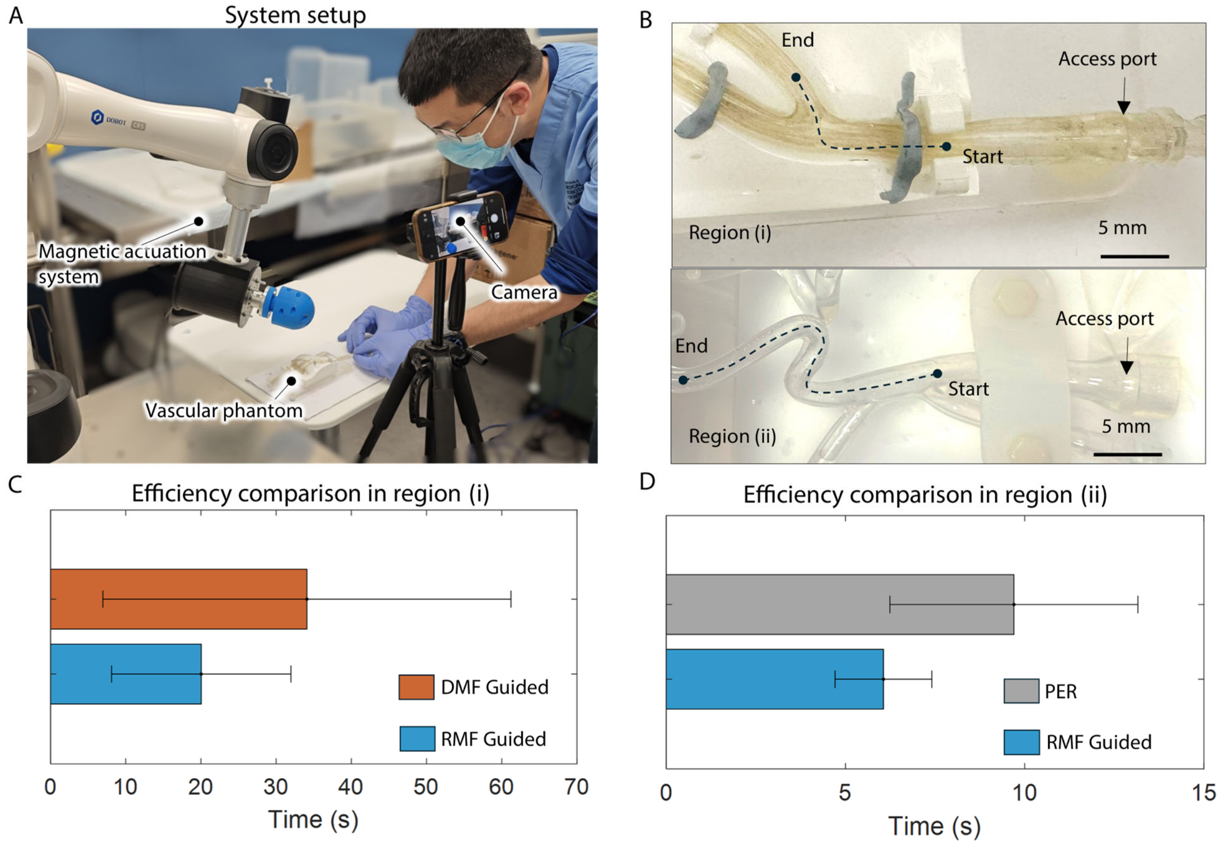

Fig. S7. Magnetic microcatheter navigation tests by an experienced operator. (A) Experiment setup. (B) Silicone model details with dashed line highlighted the desired trajectory. (C) Efficiency comparison results of navigation to region (i) using directional magnetic field (DMF) and Rotation-assisted magnetic field (RMF). The time used is from starting point to the end point. Each trial is repeated 10 times. (D) Efficiency comparison results of navigation to region (ii) using proximal end rotation (PER) and Rotation-assisted magnetic field (RMF). The time used is from starting point to the end point. Each trial is repeated 10 times.

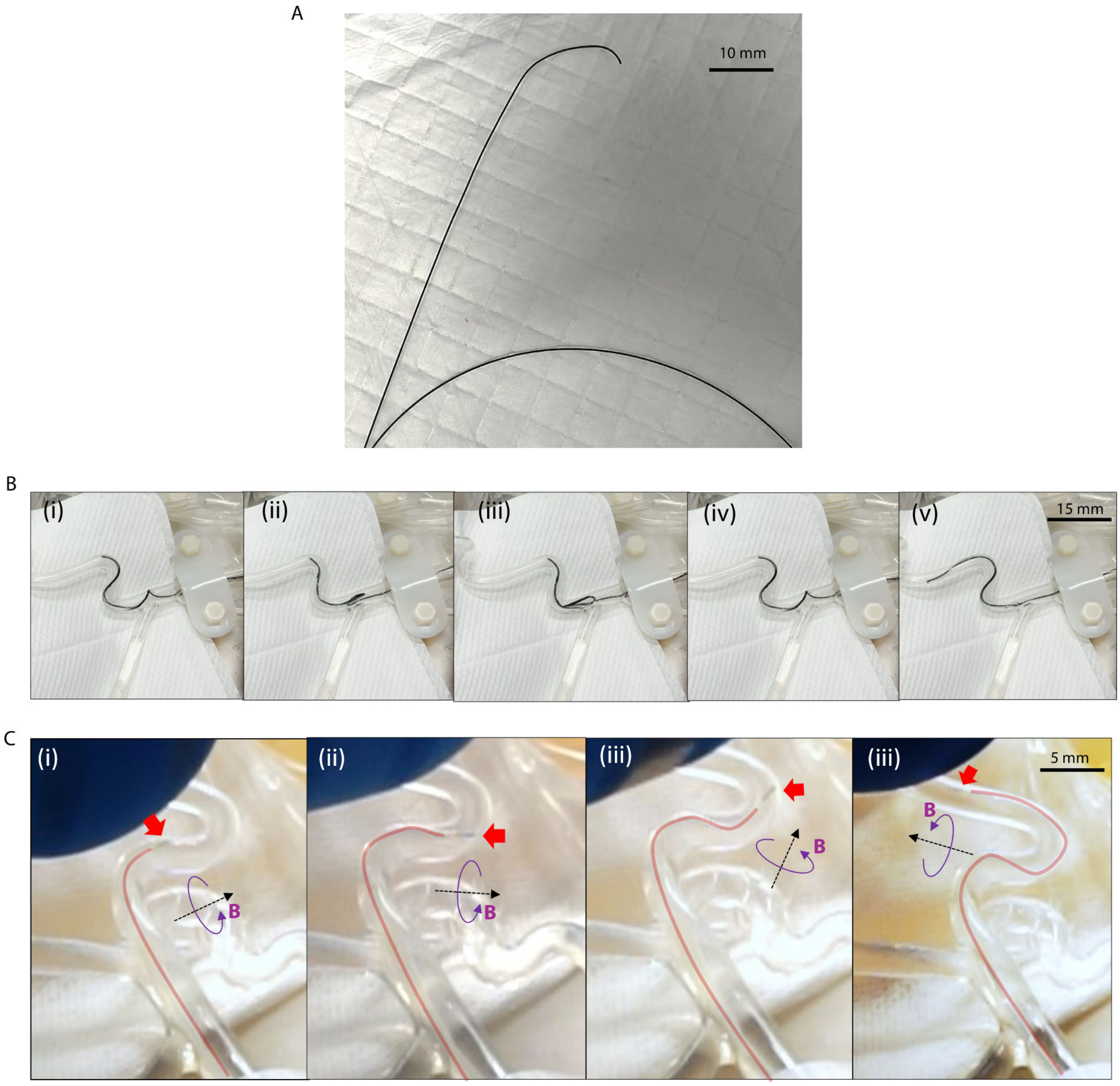

Fig. S8. Comparison between RMF guided magnetic microcatheter with conventional pre-bent tip microcatheter. (A) Pre-bent guidewire (ZIPwire™, Boston Scientific, OD: 0.89 mm). (B) Navigation using pre-bent guidewire. (C) Navigate the S-shape phantom using RMF.

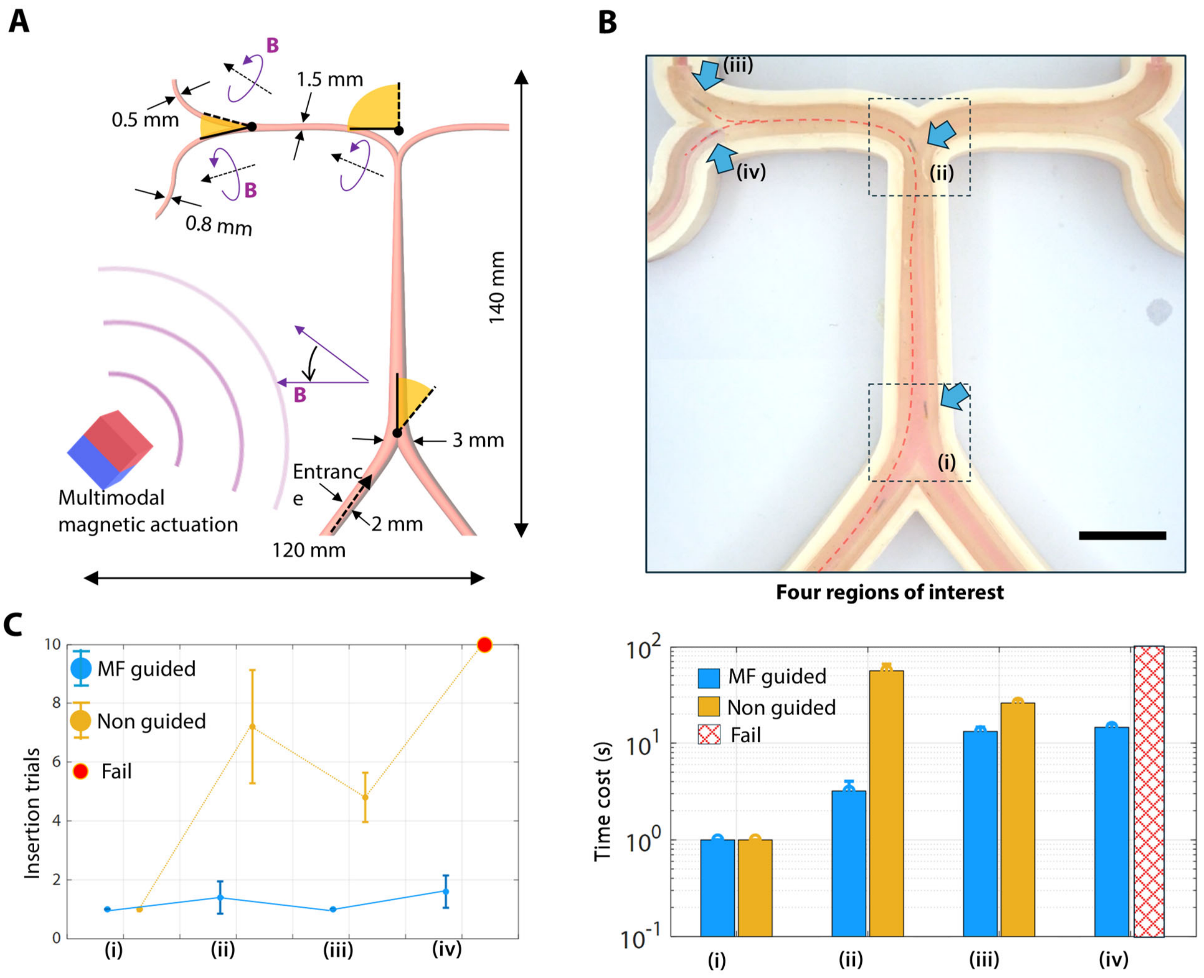

Fig. S9. Steerability evaluation after long distance navigation. (A) Navigation in a silicone phantom with features of narrow diameters and continuous sharp turns. Different magnetic navigation strategies are adopted for different angles to ensure efficiency and steerability after passing through sharp turns. (B) Key frames of magnetic navigation in the four regions of interest. The scale bar is 4 mm. (C) Quantitatively evaluate the performance of the navigation. With magnetic guidance, the navigation efficiency in terms of duration and repeated times is improved. In region (ii), where the microcatheter took a sharp turn, the magnetically guided group is 30 times faster with only 1 - 2 repeated insertions. Moreover, the magnetically guided microcatheter demonstrated selective steering ability into regions (iv) and (iii), indicating its maintained steerability after navigating acute turns while the non-guided group cannot access region (iv). In the distal branches, successful rotational actuation of the microcatheter was achieved, indicating its maintained functionality after taking sharp turns. Without magnetic guidance, the microcatheter loses steerability after passing through region (ii) and cannot enter region (iv). n = 5 samples, the error bar indicates the standard deviation.

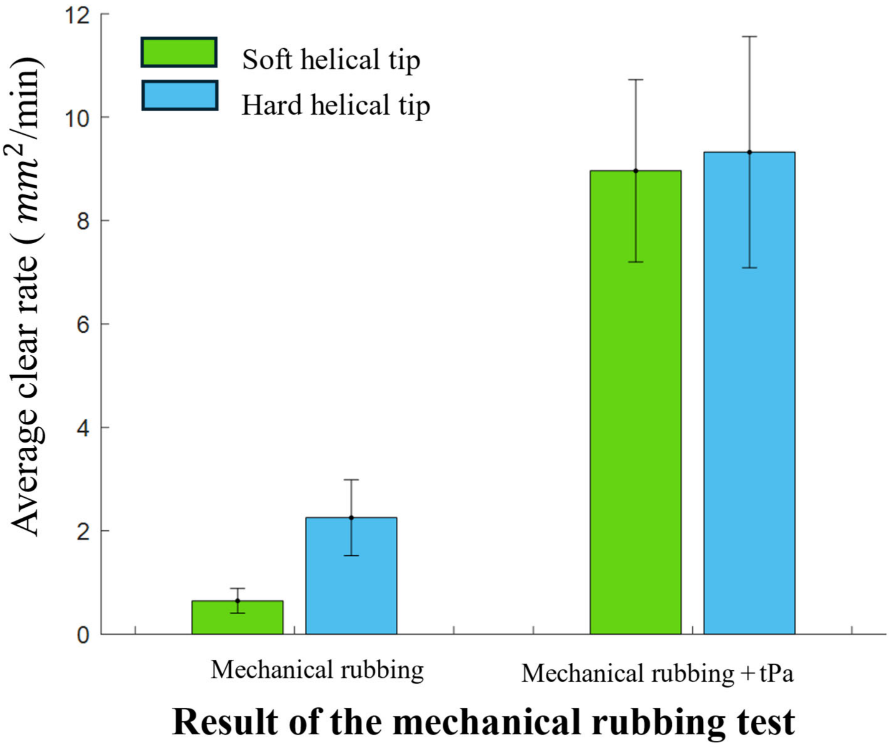

Fig. S10. Efficiency comparison between hard design and soft design. The average clearance rate of mechanical rubbing for the soft design is 28.4% of that of the hard helical tip design. However, under the assistance of the thrombolytic drug, the clearance rate of the soft design escalates to 96.13% of the hard helical tip design. The data are presented as mean values, and standard deviation for the number of trials n = 5.

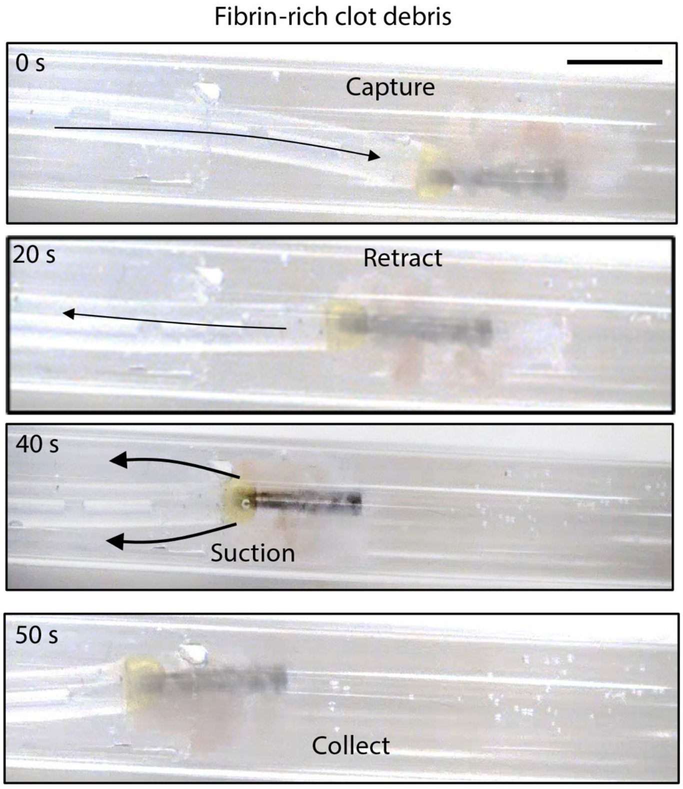

Fig. S11. Demonstration of the fibrin-rich blood clot retrieval. The scale bar is 1 mm.

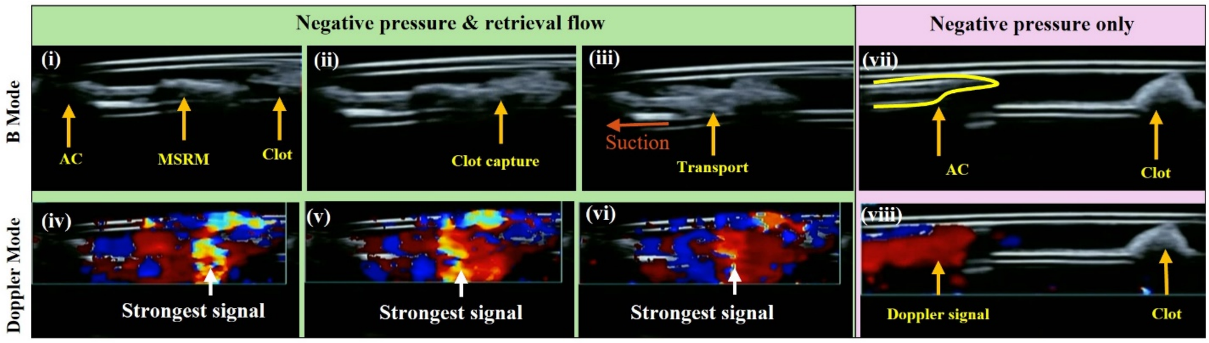

Fig. S12. Demonstration of the fibrin-rich blood clot retrieval. Retrieval artificial blood clot under the US imaging. The strong Doppler signals in the test group indicate the effective retrieval flow.

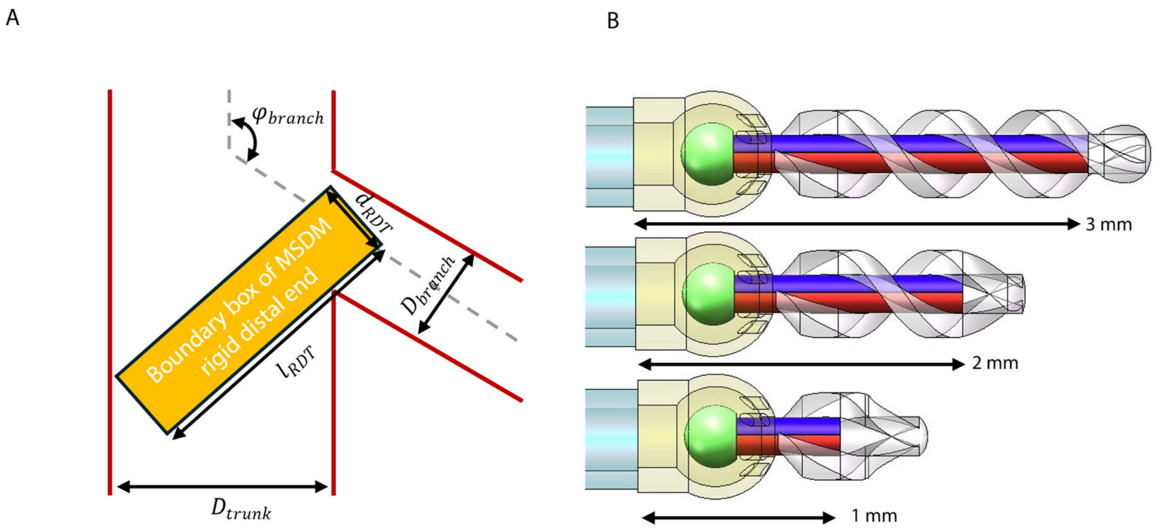

Fig. S13. Accessibility evaluation principle and downscale design. (A) 2D schematic drawing of navigating the rigid distal part from trunk artery to branch artery. (B) Downscale design for enhanced accessibility.

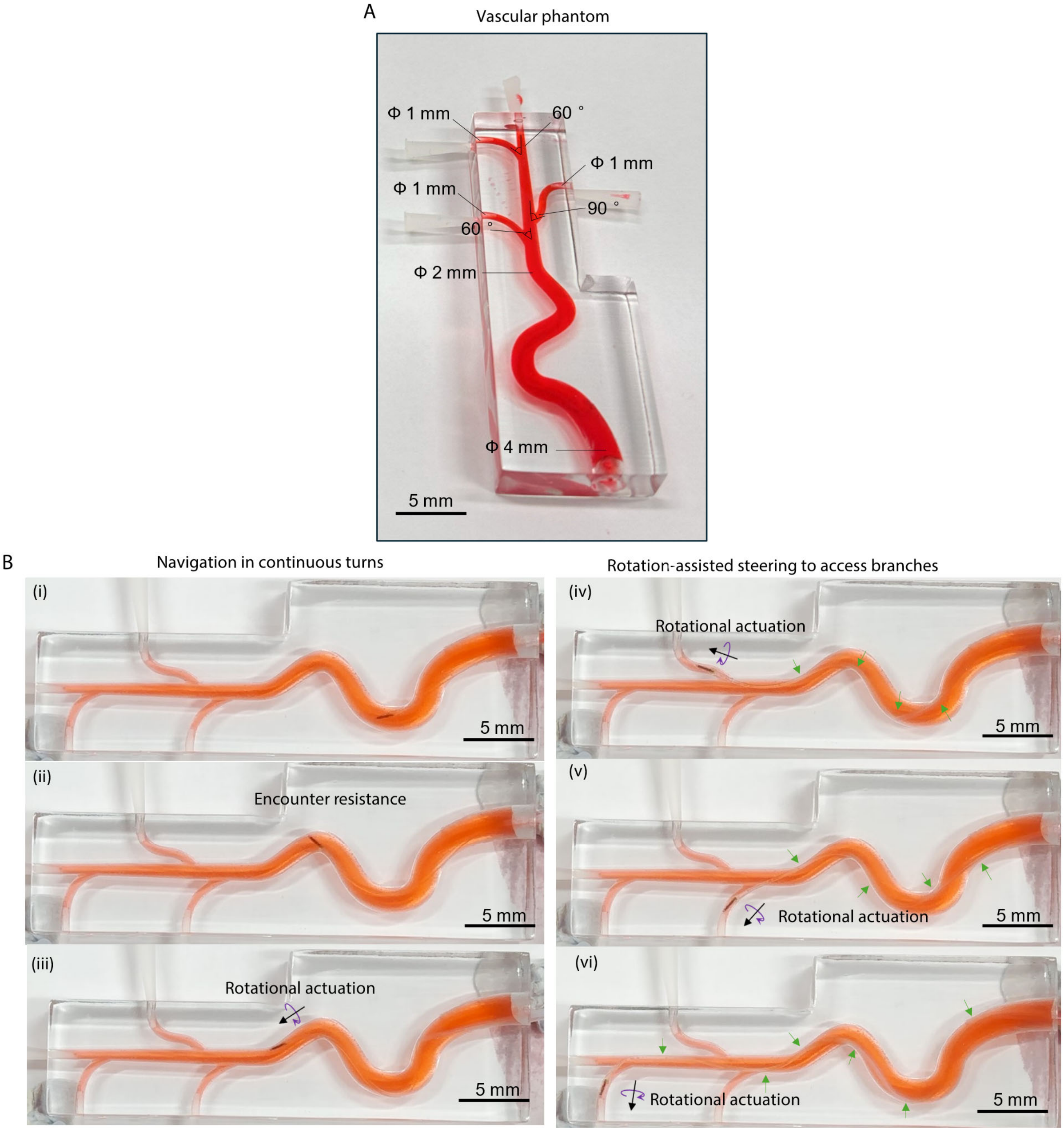

Fig. S14. Demonstration of the maintained accessibility after passing continuous turns. (A) Detailed dimensions of the vascular phantom. (B) Key frames of the navigation process (i-iii) Navigation through continuous sharp turns. (iv-vi) Access to the distal branches. Catheter body and blood vessel wall contacts are highlighted by green arrows.

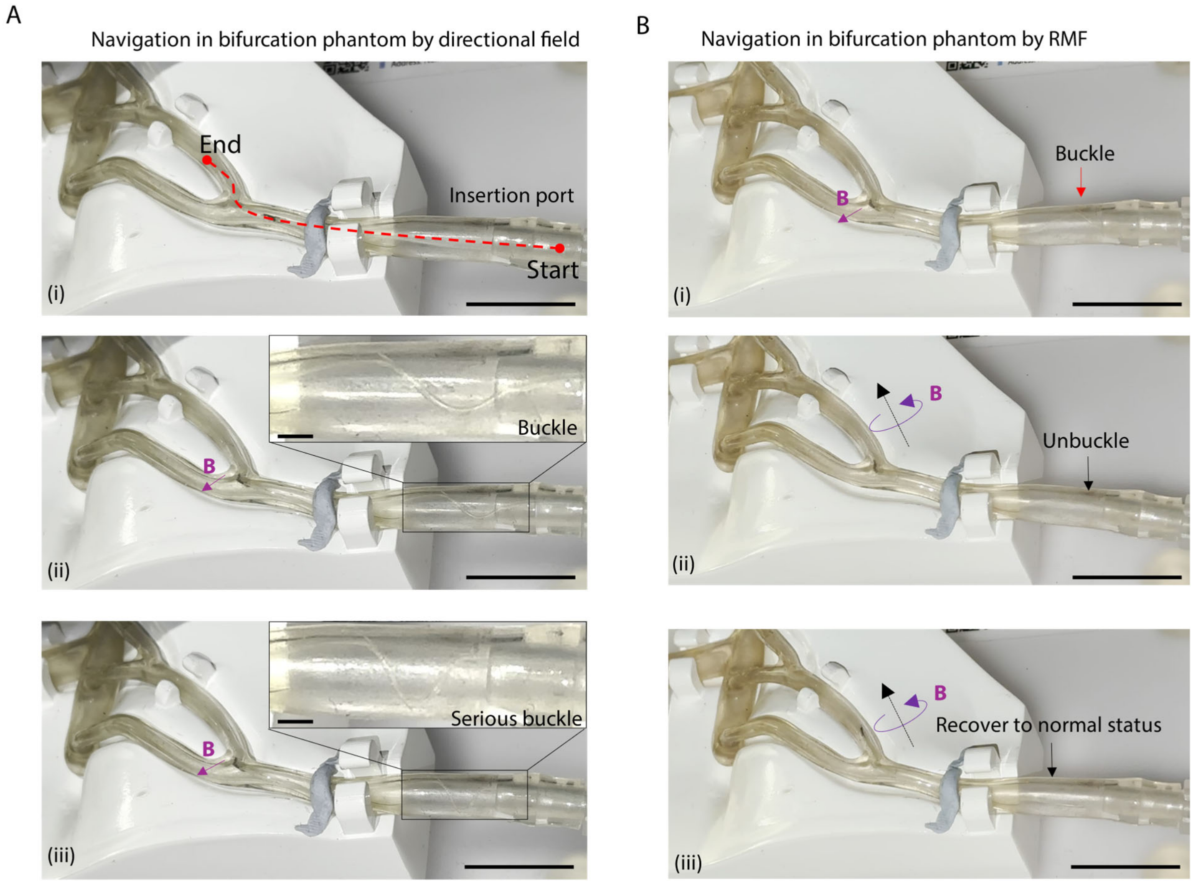

Fig. S15. Tension status evaluation in a full-size blood vessel phantom. (A) Directional magnetic field navigation results. Scale bar is 10 mm. Inset scale bar is 1 mm. (B) Rotation-assisted active steering strategy results. Scale bar is 10 mm.

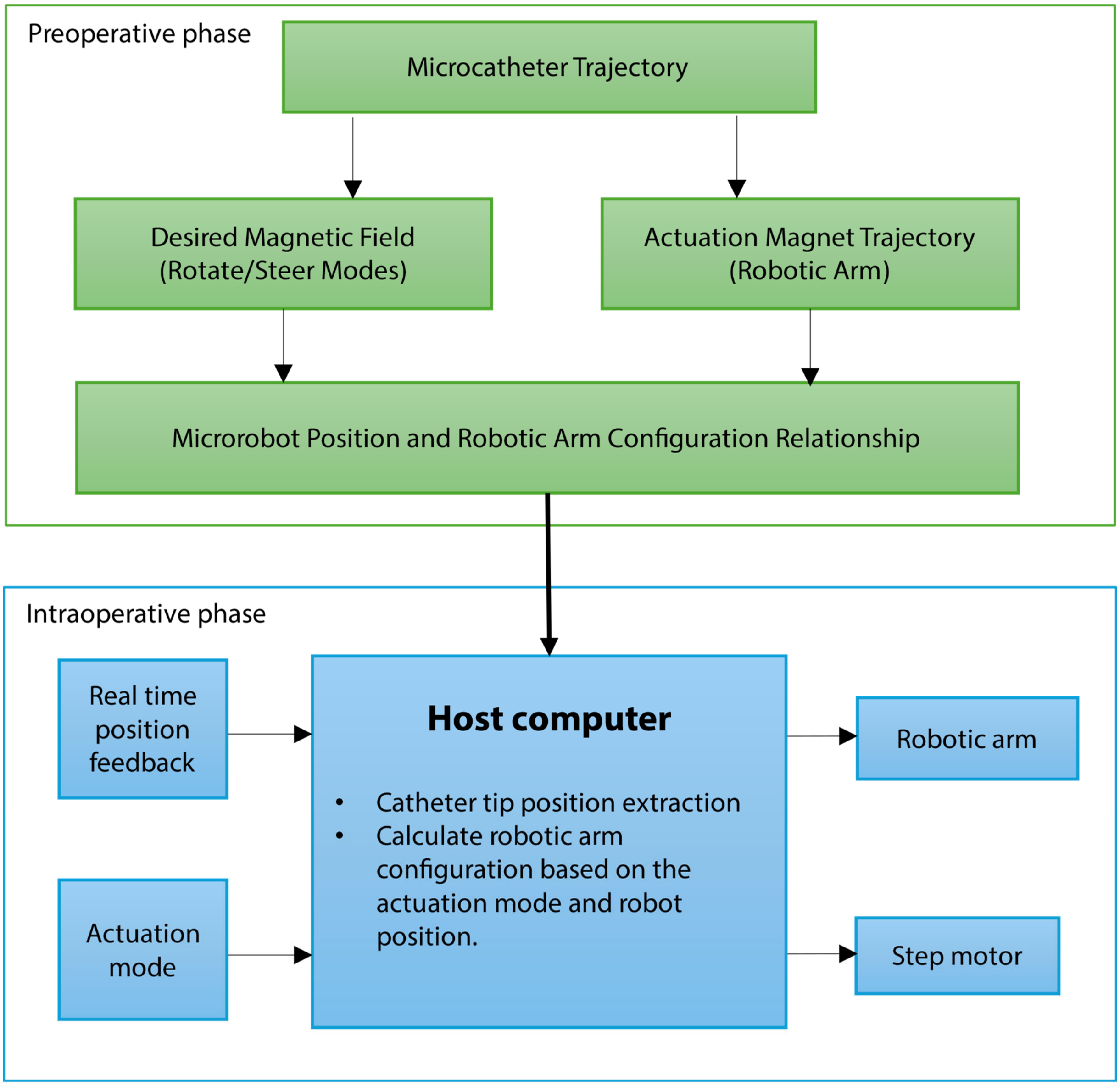

Fig. S16. Control framework of the magnetic field generation method.

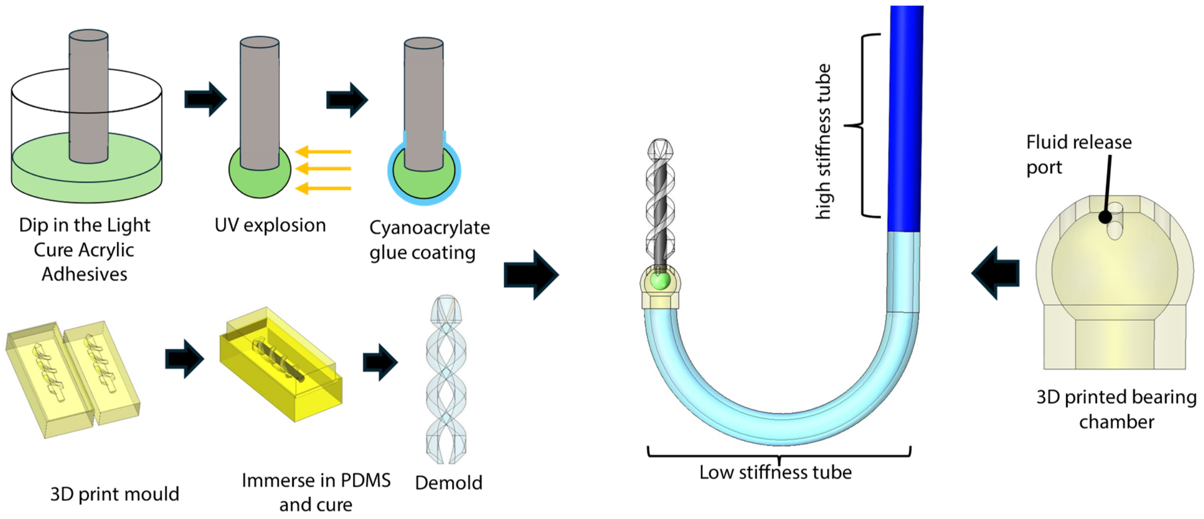

Fig. S17. Fabrication process of the MSRM. The fabrication process includes three parts: the ball joint is made from a dip and cure process; the soft helical sheath is made by negative molding technique, and the ball joint chamber is 3-D printed. Different stiffness support tubes are used for the creation of a stiffness gradient design.

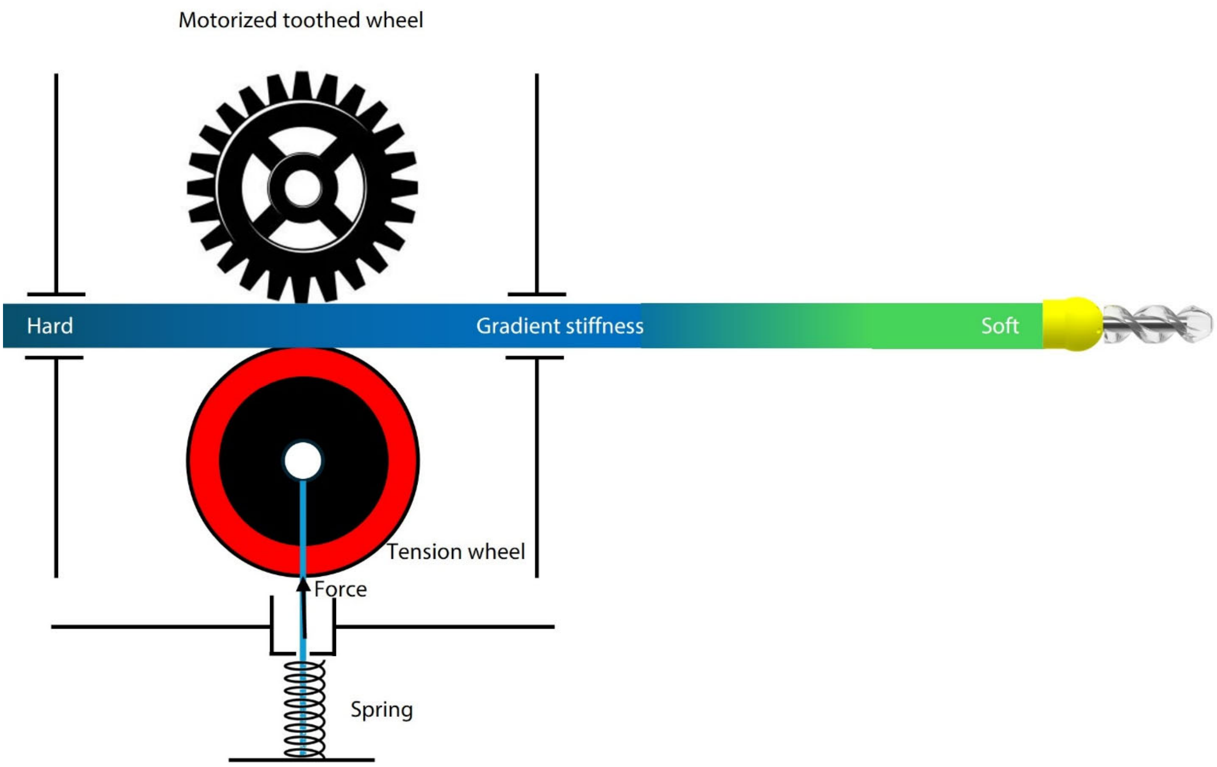

Fig. S18. Fabrication process of the MSRM. The fabrication process includes three parts: the ball joint is made from a dip and cure process; the soft helical sheath is made by negative molding technique, and the ball joint chamber is 3-D printed. Different stiffness support tubes are used for the creation of a stiffness gradient design.

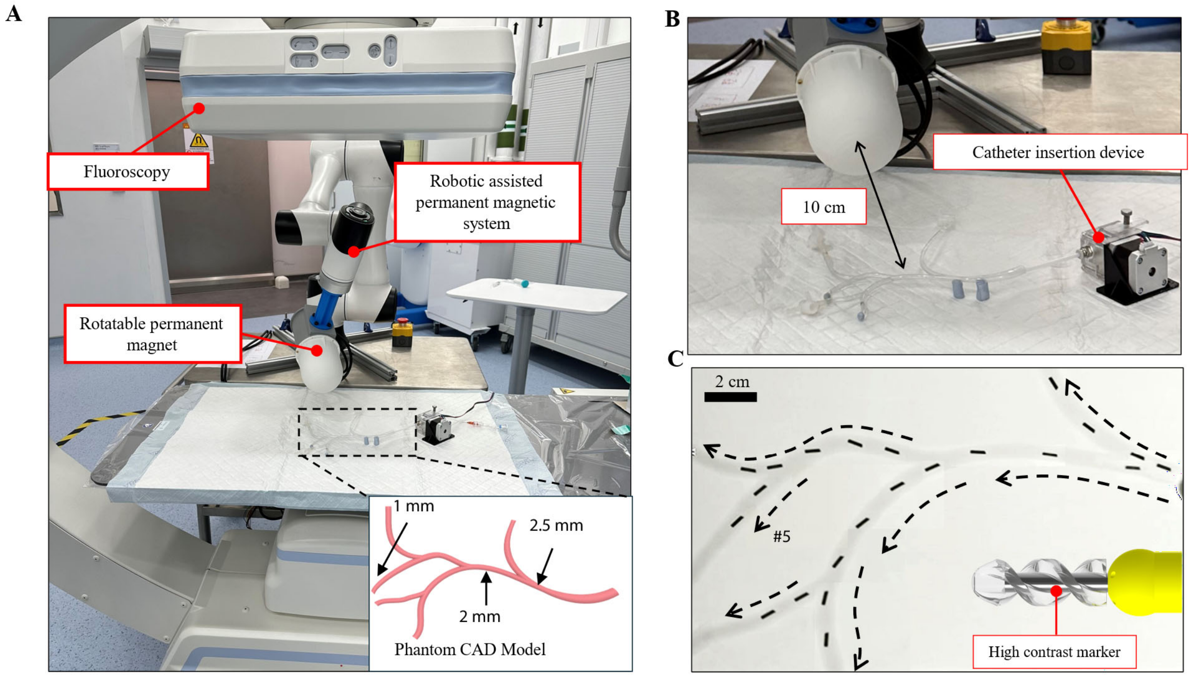

Fig. S19. System setup of the insertion performance evaluation. (A). Experiment setup of the MSRM navigation and actuation under X-ray imaging. The robotic arm carries the rotatable permanent magnetic source and shares the same workspace as the X-ray imaging system. The bifurcation phantom simulates the human brain vessel's tortuosity and dimension. (B). Safe distance demonstration and insertion machine setup. (C). The integrated navigation results. Guiding by the permanent magnetic system, the MSRM can be navigated to the desired branch. The embedded permanent magnet can be regarded as a contrast marker.

Table S1

Dimensions	Value
Tip length (l)	3 mm
Diameter (w)	0.8 mm
Ball joint chamber ID	0.5 mm
Ball joint chamber OD	0.8 mm
Soft elastomer sleeve length	2 mm
Permanent magnet OD	0.3 mm
Permanent magnet length	2.5 mm
Working channel OD	0.5 mm
Working channel ID	0.3 mm

Table S1: Detailed dimensions of helical tip design.

Table S2

**Work**	**Size (OD×Length)**	**Actuation frequency**	**Material hardness**	**Working principle**
Leclerc *et al.(23)*	2.5×6 mm (Untethered)	45 Hz	Shore hardness (D): 76 (Diamond abrasive coating)	Mechanical shear and abrasive action
Nguyen *et al.(71)*	1.6×6 mm (Untethered)	100 Hz	Shore hardness (D): 76.1 - 81.7	Mechanical shear and abrasive action
Lee *et al.(72)*	3×9 mm (Untethered)	20 - 25 Hz	Shore hardness (D): 55 - 100	Mechanical shear and abrasive action
Sa *et al.(73)*	OD: 2.1 mm (Tethered)	7 Hz	Shore hardness (D): 40	Mechanical shear and abrasive action
Yang *et al.(74)*	OD: 1.8 mm (Tethered)	8 Hz	Shore hardness (D): 93.1	Mechanical shear and abrasive action
This work	OD: 0.8 mm (Tethered)	5 - 8 Hz	Shore hardness (A): 41.7	Medicine-assisted mechanical interaction

Table S2: Comparison of untethered and tethered rotor-tipped micro machine.

Table S3

Work	Device OD	Workspace	Tip rotation	Tip softness	Multifunction
This work	0.8 mm	~270°	3D	0.1-10 MPa	5
Mao *et al.(75)*	4 mm	~360°	2D	0.1-10 MPa	4
Pittiglio *et al.(76)*	2 mm	~180°	2D	0.5–5 MPa	2
Dreyfus *et al.(30)*	0.8 mm	~270°	3D	100–300 MPa	3
Nyguyen *et al.(71)*	1.6 mm	~90°	3D	2-3 GPa	2

Table S3 Comparative table of performance across state-of-the-art magnetic catheters. Device OD refers to the microcatheter's maximum diameter, which suggests its accessibility. Workspace describes the range of bending angles the microcatheter can achieve in its application scenarios. Tip Rotation suggests the microcatheter tip's ability to rotate along the x, y, and z axes. Tip softness is determined by the Young's modulus of the microcatheter's tip. Multifunction refers to the number of integrated functions in the microcatheter. The MSRM's functions include static field active steering, rotation-assisted navigation, clot-drug interaction acceleration, mechanical thrombectomy, and clot debris retrieval.

Table S4

MCA Blood Vessel bifurcations	D_{trunk} (mm) Mean (Range)	D_{branch} (mm) Mean (Range)	φ_{branch} (°) Mean (Range)	Accessibility		
				$l_{RDT} = 3$ mm $d_{RDT} = 0.8$ mm	$l_{RDT} = 2$ mm $d_{RDT} = 0.8\ mm$	$l_{RDT} = 1$ mm $d_{RDT} = 0.8\ mm$
Superior trunk to orbitofrontal	2.29 (1.95–2.76)	1.08 (0.44–1.58)	63 (35–110)	Partial	Partial	Partial
Superior trunk to prefrontal		1.31 (1.00–1.96)		Partial	Total	Total
Superior trunk to precentral		1.49 (0.60–2.36)		Partial	Partial	Partial
Superior trunk to central		1.69 (0.86–2.47)		Partial	Total	Total
Superior trunk to postcentral		1.45 (1.04–2.20)		Partial	Total	Total
Intermediate trunk to angular	2.14 (2.03–2.20)	1.90 (1.61–2.10)	64 (30–115)	Total	Total	Total
Intermediate trunk to central		1.69 (0.86–2.47)		Partial	Total	Total
Intermediate trunk to precentral		1.49 (0.60–2.36)		Partial	Partial	Partial
Intermediate trunk to prefrontal		1.31 (1.00–1.96)		Partial	Total	Total
Inferior trunk to temporooccipital	2.55 (2.15–2.75)	1.66 (1.49–1.84)	63 (35–110)	Total	Total	Total
Inferior trunk to central		1.69 (0.86–2.47)		Partial	Total	Total
Inferior trunk to postcentral		1.45 (1.04–2.20)		Partial	Total	Total
Inferior trunk to posterior parietal		1.35 (0.82–2.00)		Partial	Total	Total
Inferior trunk to angular		1.90 (1.61–2.10)		Total	Total	Total
Temporal artery to Temporooccipital	1.74 (1.57–1.83)	1.66 (1.49–1.84)	72 (50–130)	Partial	Total	Total
Temporal artery to posterior temporal		1.34 (1.24–1.44)		Partial	Partial	Total
Temporal artery to temporopolar		0.90 (0.82–1.32)		Not applicable	Partial	Total

Table S4. Evaluation of the proposed microcatheter's accessibility in MCA bifurcations.

Table S5

Microcatheter	Application	Diameter	Rigid tip length
Dreyfus *et al.(30)*	Access to neurovascular system	0.8 mm	~ 3.2 mm
Zhang *et al.(77)*	Intravascular OCT imaging	0.89 mm	6 mm
Wang *et al.(78)*	Intravascular ultrasound imaging	1.3 mm	~ 2.2 mm
Nyguyen *et al.(71)*	Mechanical atherectomy	2 mm	> 5 mm
The proposed work	Blood clot treatment	0.8 mm	3 mm

Table S5. Rigid distal tip comparison between the state-of-the-art microcatheter for distal vascular.

Table S6

Description	Value
Permanent Magnetic Manipulation System	5-DoF Robotic-arm assisted permanent magnet
Robotic Arm	Dobot CR5, Shenzhen Yuejiang Technology Co., Ltd.
Cubic Permanent Size	50 mm
X-ray Imaging Device	Siemens Artis Zeego C-Arm X-ray
Settper Motors	Leadshine, 57cme23.

Table S6: Hardware details of the permanent magnetic system.

Movie S1.
Steerability tests in the branch phantom. The video first demonstrates the comparison of rotation-assisted method and directional magnetic field guided method and then shows a full branch navigation.

Movie S2.
Rotation-assisted navigation force analysis.

Movie S3.
Pumping effect demonstration.

Movie S4.
Magnetic navigation in life-size silicone vascular phantom: Bifurcations from M3 to M4.

Movie S5.
Magnetic navigation in life-size silicone vascular phantom: S-shape vascular of ICA.

Movie S6.
In vitro accessibility verification test.

Movie S7.
In vitro thrombosis treatment demonstration.

Movie S8.
Blood clot debris retrieval demonstration.

Movie S9.
Ultrasound imaging guided clot debris retrieval.

Movie S10.
X-ray imaging guided navigation in vivo.

Movie S11.
Accessibility evaluation after continuous turns.

Movie S12.
Tension status evaluation and efficiency comparison between DMF and RMF.

REFERENCES AND NOTES

1. J. L. Saver, Time is brain—Quantified. *Stroke* **37**, 263–266 (2006).

2. G. De Luca, H. Suryapranata, J. P. Ottervanger, E. M. Antman, Time delay to treatment and mortality in primary angioplasty for acute myocardial infarction: Every minute of delay counts. *Circulation* **109**, 1223–1225 (2004).

3. D. Sepp, M. R. Hernandez Petzsche, T. Zarth, S. Wunderlich, B. Ikenberg, C. Maegerlein, C. Zimmer, M. T. Berndt, T. Boeckh-Behrens, J. S. Kirschke, Mechanical thrombectomy of distal cerebral vessel occlusions of the anterior circulation. *Sci. Rep.* **13**, 5730 (2023).

4. M. C. Berger, A. Simgen, P. Dietrich, W. Naziri, Safety and efficacy of thrombectomy for distal medium vessel occlusions of the middle cerebral artery. *Neurointervention* **20**, 15–23 (2025).

5. D. Schartz, N. Ellens, G. S. Kohli, R. Rahmani, S. M. K. Akkipeddi, G. P. Colby, F. Hui, T. Bhalla, T. Mattingly, M. T. Bender, Impact of aspiration catheter size on clinical outcomes in aspiration thrombectomy. *J. Neurointerv. Surg.* **15**, e111–e116 (2023).

6. D. R. Santiago-Dieppa, J. Friend, Endovascular microrobotics for neurointervention. *Annu. Rev. Control Robot. Auton. Syst.* **7**, 385–408 (2024).

7. A. von Hessling, T. Reyes del Castillo, G. Karwacki, J. E. Roos, The Columbus steerable guidewire in neurointerventions: Early clinical experience and applications. *J. Neurointerv. Surg.* **14**, 291–296 (2022).

8. F. Maisano, H. Vanermen, J. Seeburger, M. Mack, V. Falk, P. Denti, M. Taramasso, O. Alfieri, Direct access transcatheter mitral annuloplasty with a sutureless and adjustable device: Preclinical experience. *Eur. J. Cardiothorac. Surg.* **42**, 524–529 (2012).

9. K. Tiroch, M. Vorpahl, M. Seyfarth, Novel mitral clipping technique overcoming extreme atrial dilatation. *Catheter. Cardiovasc. Interv.* **84**, 606–609 (2014).

10. J. Joseph, K. C. K. Wong, M. R. Ginks, Y. Bashir, T. R. Betts, K. Rajappan, Steerable sheath technology in the ablation of atrial fibrillation. *Recent Pat. Cardiovasc. Drug Discov.* **8**, 171–177 (2013).

11. H. Rafii-Tari, C. J. Payne, G.-Z. Yang, Current and emerging robot-assisted endovascular catheterization technologies: A review. *Ann. Biomed. Eng.* **42**, 697–715 (2014).

12. W. Lee, J. Nam, J. Kim, E. Jung, N. Kim, G. Jang, Steering, tunneling, and stent delivery of a multifunctional magnetic catheter robot to treat occlusive vascular disease. *IEEE Trans. Ind. Electron.* **68**, 391–400 (2021).

13. J. M. Wardlaw, C. Smith, M. Dichgans, Small vessel disease: Mechanisms and clinical implications. *Lancet Neurol.* **18**, 684–696 (2019).

14. Y. Dong, L. Wang, V. Iacovacci, X. Wang, L. Zhang, B. J. Nelson, Magnetic helical micro-/nanomachines: Recent progress and perspective. *Matter* **5**, 77–109 (2022).

15. Y. Kim, E. Genevriere, P. Harker, J. Choe, M. Balicki, R. W. Regenhardt, J. E. Vranic, A. A. Dmytriw, A. B. Patel, X. Zhao, Telerobotic neurovascular interventions with magnetic manipulation. *Sci. Robot.* **7**, eabg9907 (2022).

16. Z. Yang, L. Yang, M. Zhang, N. Xia, L. Zhang, Ultrasound-guided wired magnetic microrobot with active steering and ejectable tip. *IEEE Trans. Ind. Electron.* **70**, 614–623 (2023).

17. Z. Yang, H. Yang, Y. Cao, Y. Cui, L. Zhang, Magnetically actuated continuum medical robots: A review. *Adv. Intell. Syst.* **5**, 2200416 (2023).

18. J. Hu, S. Huang, L. Zhu, W. Huang, Y. Zhao, K. Jin, Q. ZhuGe, Tissue plasminogen activator-porous magnetic microrods for targeted thrombolytic therapy after ischemic stroke. *ACS Appl. Mater. Interfaces* **10**, 32988–32997 (2018).

19. T. Wang, H. Ugurlu, Y. Yan, M. Li, M. Li, A.-M. Wild, E. Yildiz, M. Schneider, D. Sheehan, W. Hu, M. Sitti, Adaptive wireless millirobotic locomotion into distal vasculature. *Nat. Commun.* **13**, 4465 (2022).

20. X. Tang, L. Manamanchaiyaporn, Q. Zhou, C. Huang, L. Li, Z. Li, L. Wang, J. Wang, L. Ren, T. Xu, X. Yan, Y. Zheng, Synergistic integration and pharmacomechanical function of enzyme-magnetite nanoparticle swarms for low-dose fast thrombolysis. *Small* **18**, e2202848 (2022).

21. H. Xu, M. Medina-Sánchez, M. F. Maitz, C. Werner, O. G. Schmidt, Sperm micromotors for cargo delivery through flowing blood. *ACS Nano* **14**, 2982–2993 (2020).

22. A. V. Pozhitkova, D. V. Kladko, D. A. Vinnik, S. V. Taskaev, V. V. Vinogradov, Reprogrammable soft swimmers for minimally invasive thrombus extraction. *ACS Appl. Mater. Interfaces* **14**, 23896–23908 (2022).

23. J. Leclerc, H. Zhao, D. Bao, A. T. Becker, In vitro design investigation of a rotating helical magnetic swimmer for combined 3-D navigation and blood clot removal. *IEEE Trans. Robot.* **36**, 975–982 (2020).

24. B. J. Nelson, S. Gervasoni, P. W. Y. Chiu, L. Zhang, A. Zemmar, Magnetically actuated medical robots: An in vivo perspective. *Proc. IEEE* **110**, 1028–1037 (2022).

25. Y. Kim, G. A. Parada, S. Liu, X. Zhao, Ferromagnetic soft continuum robots. *Sci. Robot.* **4**, eaax7329 (2019).

26. Y. Piskarev, J. Shintake, C. Chautems, J. Lussi, Q. Boehler, B. J. Nelson, D. Floreano, A variable stiffness magnetic catheter made of a conductive phase-change polymer for minimally invasive surgery. *Adv. Funct. Mater.* **32**, 2107662 (2022).

27. L. Pancaldi, P. Dirix, A. Fanelli, A. M. Lima, N. Stergiopulos, P. J. Mosimann, D. Ghezzi, M. S. Sakar, Flow driven robotic navigation of microengineered endovascular probes. *Nat. Commun.* **11**, 6356 (2020).

28. J. Edelmann, A. J. Petruska, B. J. Nelson, Magnetic control of continuum devices. *Int. J. Rob. Res.* **36**, 68–85 (2017).

29. A. J. Sperry, T. J. Schwehr, E. K. Pinegar, O. B. Richards, J. D. Rolston, M. D. Alexander, B. Coats, J. J. Abbott, A. Kuntz, Screw-tip soft magnetically steerable needles. *IEEE Trans. Med. Robot. Bionics* **6**, 4–17 (2024).

30. R. Dreyfus, Q. Boehler, S. Lyttle, P. Gruber, J. Lussi, C. Chautems, S. Gervasoni, J. Berberat, D. Seibold, N. Ochsenbein-Kölble, M. Reinehr, M. Weisskopf, L. Remonda, B. J. Nelson, Dexterous helical magnetic robot for improved endovascular access. *Sci. Robot.* **9**, eadh0298 (2024).

31. D. von Arx, C. Fischer, H. Torlakcik, S. Pané, B. J. Nelson, Q. Boehler, Simultaneous localization and actuation using electromagnetic navigation systems. *IEEE Trans. Robot.* **40**, 1292–1308 (2024).

32. Y. Yan, T. Wang, R. Zhang, Y. Liu, W. Hu, M. Sitti, Magnetically assisted soft milli-tools for occluded lumen morphology detection. *Sci. Adv.* **9**, eadi3979 (2023).

33. C. M. Heunis, K. J. Behrendt, E. E. G. Hekman, C. Moers, J.-P. P. M. de Vries, S. Misra, Design and evaluation of a magnetic rotablation catheter for arterial stenosis. *IEEE/ASME Trans. Mechatron.* **27**, 1761–1772 (2022).

34. H. Yang, Z. Yang, D. Jin, L. Su, K. F. Chan, K. K. L. Chong, C. P. Pang, L. Zhang, Magnetic micro-driller system for nasolacrimal duct recanalization. *IEEE Robot. Autom. Lett.* **7**, 7367–7374 (2022).

35. F. Settecase, M. S. Sussman, M. W. Wilson, S. Hetts, R. L. Arenson, V. Malba, A. F. Bernhardt, W. Kucharczyk, T. P. L. Roberts, Magnetically-assisted remote control (MARC) steering of endovascular catheters for interventional MRI: A model for deflection and design implications. *Med. Phys.* **34**, 3135–3142 (2007).

36. B. Zhang, H. Wu, H. Kim, P. J. Welch, A. Cornett, G. Stocker, R. G. Nogueira, J. Kim, G. Owens, P. A. Dayton, Z. Xu, C. Shi, X. Jiang, A model of high-speed endovascular sonothrombolysis with vortex ultrasound-induced shear stress to treat cerebral venous sinus thrombosis. *Research (Wash D C)* **6**, 0048 (2023).

37. R. Gupta, Risks of microcatheter injections in acute stroke treatment. *Nat. Rev. Neurol.* **5**, 181–182 (2009).

38. J. L. Saver, R. Chapot, R. Agid, A. Hassan, A. P. Jadhav, D. S. Liebeskind, K. Lobotesis, D. Meila, L. Meyer, G. Raphaeli, R. Gupta, Distal Thrombectomy Summit Group, Thrombectomy for distal, medium vessel occlusions: A consensus statement on present knowledge and promising directions. *Stroke* **51**, 2872–2884 (2020).

39. K. Takashima, R. Shimomura, T. Kitou, H. Terada, K. Yoshinaka, K. Ikeuchi, Contact and friction between catheter and blood vessel. *Tribol. Int.* **40**, 319–328 (2007).

40. S. Wiegand, Thermal diffusion in liquid mixtures and polymer solutions. *J. Phys. Condens. Matter* **16**, R357 (2004).

41. P. Riha, X. Wang, R. Liao, J. F. Stoltz, Elasticity and fracture strain of whole blood clots. *Clin. Hemorheol. Microcirc.* **21**, 45-49 (1999).

42. Z. Wang, K. Wang, Y. Xu, Friction injury of the central vein caused by catheter for hemodialysis: An in vitro study. *Sci. Rep.* **14**, 5836 (2024).

43. M. Abbasi, J. Arturo Larco, M. O. Mereuta, Y. Liu, S. Fitzgerald, D. Dai, R. Kadirvel, L. Savastano, D. F. Kallmes, W. Brinjikji, Diverse thrombus composition in thrombectomy stroke patients with longer time to recanalization. *Thromb. Res.* **209**, 99–104 (2022).

44. E. Mfoumou, J. Tripette, M. Blostein, G. Cloutier, Time-dependent hardening of blood clots quantitatively measured in vivo with shear-wave ultrasound imaging in a rabbit model of venous thrombosis. *Thromb. Res.* **133**, 265–271 (2014).

45. Y. Chu, H. Liu, P. Xing, G. Lou, C. Wu, The morphology and haemodynamics of the rabbit renal artery: Evaluation by conventional and contrast-enhanced ultrasonography. *Lab. Anim* **45**, 204–208 (2011).

46. D. Lin, N. Jiao, Z. Wang, L. Liu, A magnetic continuum robot with multi-mode control using opposite-magnetized magnets. *IEEE Robot. Autom. Lett.* **6**, 2485–2492 (2021).

47. D. Lin, J. Wang, N. Jiao, Z. Wang, L. Liu, A flexible magnetically controlled continuum robot steering in the enlarged effective workspace with constraints for retrograde intrarenal surgery. *Adv. Intell. Syst.* **3**, 2000211 (2021).

48. J. Li, L. Wang, Modeling magnetic soft continuum robot in nonuniform magnetic fields via energy minimization. *Int. J. Mech. Sci.* **282**, 109688 (2024).

49. C. Zhou, Y. Yang, J. Wang, Q. Wu, Z. Gu, Y. Zhou, X. Liu, Y. Yang, H. Tang, Q. Ling, L. Wang, J. Zang, Ferromagnetic soft catheter robots for minimally invasive bioprinting. *Nat. Commun.* **12**, 5072 (2021).

50. H. Torlakcik, S. Sevim, P. Alves, M. Mattmann, J. Llacer-Wintle, M. Pinto, R. Moreira, A. D. Flouris, F. C. Landers, X.-Z. Chen, J. Puigmartí-Luis, Q. Boehler, T. S. Mayor, M. Kim, B. J. Nelson, S. Pané, Magnetically guided microcatheter for targeted injection of magnetic particle swarms. *Adv. Sci.* **11**, e2404061 (2024).

51. W. Yang, C. Zhang, H. Dai, C. Hu, X. Xia, A novel wireless 5-D electromagnetic tracking system based on nine-channel sinusoidal signals. *IEEE/ASME Trans. Mech.* **26**, 246–254 (2020).

52. K. Wang, X. Wang, J. D.-L. Ho, G. Fang, B. Zhu, R. Xie, Y.-H. Liu, K. W. S. Au, J. Y.-K. Chan, K.-W. Kwok, A fast soft robotic laser sweeping system using data-driven modeling approach. *IEEE Trans. Robot.* **39**, 3043–3058 (2023).

53. L. Zhang, C. Li, H. Dong, X. Liu, T. Sun, K. T. V. Grattan, J. Zhao, Fiber Bragg Grating-based sensor system for sensing the shape of flexible needles. *Measurement* **206**, 112251 (2023).

54. S. Yuan, C. Xu, B. Cui, T. Zhang, B. Liang, W. Yuan, H. Ren, Motor-free telerobotic endomicroscopy for steerable and programmable imaging in complex curved and localized areas. *Nat. Commun.* **15**, 7680 (2024).

55. T. Zhang, G. Li, H. Ren, L. Yang, X. Yang, R. Tan, Y. Tang, D. Guo, H. Zhao, W. Shang, Y. Shen, Sub-millimeter fiberscopic robot with integrated maneuvering, imaging, and biomedical operation abilities. *Nat. Commun.* **15**, 10874 (2024).

56. X. Yang, W. Shang, H. Lu, Y. Liu, L. Yang, R. Tan, X. Wu, Y. Shen, An agglutinate magnetic spray transforms inanimate objects into millirobots for biomedical applications. *Sci. Robot.* **5**, eabc8191 (2020).

57. Z. He, J. Dai, J. D.-L. Ho, H.-S. Tong, X. Wang, G. Fang, L. Liang, C.-L. Cheung, Z. Guo, H.-C. Chang, I. Iordachita, R. H. Taylor, W.-S. Poon, D. T.-M. Chan, K.-W. Kwok, Interactive multi-stage robotic positioner for intra-operative MRI-guided stereotactic neurosurgery. *Adv. Sci.* **11**, e2305495 (2024).

58. J. Sikorski, C. M. Heunis, R. Obeid, V. K. Venkiteswaran, S. Misra, A flexible catheter system for ultrasound-guided magnetic projectile delivery. *IEEE Trans. Robot.* **38**, 1959–1972 (2022).

59. G. Kahilogullari, H. C. Ugur, A. Comert, I. Tekdemir, Y. Kanpolat, The branching pattern of the middle cerebral artery: Is the intermediate trunk real or not? An anatomical study correlating with simple angiography. *J. Neurosurg.* **116**, 1024–1034 (2012).

60. C. Brandt-Wunderlich, N. Grabow, K.-P. Schmitz, S. Siewert, W. Schmidt, Cardiovascular catheter stiffness—A static measurement approach. *Curr. Dir. Biomed. Eng.* **7**, 721–723 (2021).

61. B. Fereidoonnezhad, A. Dwivedi, S. Johnson, R. McCarthy, P. McGarry, Blood clot fracture properties are dependent on red blood cell and fibrin content. *Acta Biomater.* **127**, 213–228 (2021).

62. C. Maegerlein, S. Prothmann, K. E. Lucia, C. Zimmer, B. Friedrich, J. Kaesmacher, Intraprocedural thrombus fragmentation during interventional stroke treatment: A comparison of direct thrombus aspiration and stent retriever thrombectomy. *Cardiovasc. Intervent. Radiol.* **40**, 987–993 (2017).

63. L. Villalba, R. Deen, B. Tonson-Older, C. Costello, Single-session catheter-directed lysis using adjunctive clot fragmentation with power pulse spray only is a fast, safe, and effective

option for acute pulmonary embolism. *J. Vasc. Surg. Venous Lymphat. Disord.* **12**, 101899 (2024).

64. Q. Wang, B. Wang, K. F. Chan, X. Song, Q. Wang, F. Ji, L. Su, B. Y. M. Ip, H. Ko, P. W. Y. Chiu, T. W. H. Leung, L. Zhang, Rapid blood clot removal via remote delamination and magnetization of clot debris. *Adv. Sci.* **12**, 2415305 (2025).

65. B. Wang, Q. Wang, K. F. Chan, Z. Ning, Q. Wang, F. Ji, H. Yang, S. Jiang, Z. Zhang, B. Y. M. Ip, H. Ko, J. P. W. Chung, M. Qiu, J. Han, P. W. Y. Chiu, J. J. Y. Sung, S. Du, T. W. H. Leung, S. C. H. Yu, L. Zhang, tPA-anchored nanorobots for in vivo arterial recanalization at submillimeter-scale segments. *Sci. Adv.* **10**, eadk8970 (2024).

66. T. Hölscher, D. J. Fisher, G. Ahadi, A. Voie, Introduction of a rabbit carotid artery model for sonothrombolysis research. *Transl. Stroke Res.* **3**, 397–407 (2012).

67. D. E. Carter, T. Peng, M. R. Moody, S. L. Huang, D. D. McPherson, M. E. Klegerman, An echogenic clot method for thrombolysis monitoring in thrombotic stroke models. *Med. Res. Arch.* **11**, 3702 (2023).

68. Y. Kuroiwa, A. Yamashita, T. Miyati, E. Furukoji, M. Takahashi, T. Azuma, H. Sugimura, T. Asanuma, S. Tamura, K. Kawai, Y. Asada, MR signal change in venous thrombus relates organizing process and thrombolytic response in rabbit. *Magn. Reson. Imaging* **29**, 975–984 (2011).

69. T. Toyoda, K. Isobe, T. Tsujino, Y. Koyata, F. Ohyagi, T. Watanabe, M. Nakamura, Y. Kitamura, H. Okudera, K. Nakata, T. Kawase, Direct activation of platelets by addition of $CaCl_2$ leads coagulation of platelet-rich plasma. *Int. J. Implant Dent.* **4**, 23 (2018).

70. A. N. Das, R. Murthy, D. O. Popa, H. E. Stephanou, A multiscale assembly and packaging system for manufacturing of complex micro-nano devices. *IEEE Trans. Autom. Sci. Eng.* **9**, 160–170 (2011).

71. K. T. Nguyen, S.-J. Kim, H.-K. Min, M. C. Hoang, G. Go, B. Kang, J. Kim, E. Choi, A. Hong, J.-O. Park, C.-S. Kim, Guide-wired helical microrobot for percutaneous

revascularization in chronic total occlusion in-vivo validation. *IEEE Trans. Biomed. Eng.* **68**, 2490–2498 (2021).

72. S. Lee, S. Lee, S. Kim, C.-H. Yoon, H.-J. Park, J.-y. Kim, H. Choi, Fabrication and characterization of a magnetic drilling actuator for navigation in a three-dimensional phantom vascular network. *Sci. Rep.* **8**, 3691 (2018).

73. J. Sa, J. Park, E. Jung, N. Kim, D. Lee, S. Bae, Y. Lee, G. Jang, Separable and recombinable magnetic robot for robotic endovascular intervention. *IEEE Robot. Autom. Lett.* **8**, 1881–1888 (2023).

74. Z. Yang, L. Yang, M. Zhang, C. Zhang, S. C. H. Yu, L. Zhang, Ultrasound-guided catheterization using a driller-tipped guidewire with combined magnetic navigation and drilling motion. *IEEE/ASME Trans. Mech.* **27**, 2829–2840 (2022).

75. L. Mao, P. Yang, C. Tian, X. Shen, F. Wang, H. Zhang, X. Meng, H. Xie, Magnetic steering continuum robot for transluminal procedures with programmable shape and functionalities. *Nat. Commun.* **15**, 3759 (2024).

76. G. Pittiglio, P. Lloyd, T. da Veiga, O. Onaizah, C. Pompili, J. H. Chandler, P. Valdastri, Patient-specific magnetic catheters for atraumatic autonomous endoscopy. *Soft Robot.* **9**, 1120–1133 (2022).

77. C. Zhang, F. Yang, F. Wang, Q. Tan, H. He, S. Zhang, J. Wu, S. Tu, High-speed submillimeter magnetic-driven rotational scanning side-imaging OCT probe. *J. Biophotonics* **16**, e202300106 (2023).

78. L. Wang, Y. Zhao, B. Zheng, Y. Huo, Y. Fan, D. Ma, Y. Gu, P. Wang, Ultrawide-bandwidth high-resolution all-optical intravascular ultrasound using miniaturized photoacoustic transducer. *Sci. Adv.* **9**, eadg8600 (2023).
